# L013 promotes NRF2 degradation in NRFA-subtype esophageal squamous cell carcinoma

**DOI:** 10.1016/j.xcrm.2026.103046

**Published:** 2026-09-11

**Authors:** Xiao Yu, Yue Wu, Xiuyun Sun, Rui Han, Yi Cai, Wei Zeng, Yang Li, Yuhao Wang, Yahui Zhao, Yu Rao, Zhihua Liu

**Affiliations:** 1 State Key Laboratory of Molecular Oncology, National Cancer Center/National Clinical Research Center for Cancer/Cancer Hospital, Chinese Academy of Medical Sciences and Peking Union Medical College, Beijing 100021, China; 2 MOE Key Laboratory of Protein Sciences, School of Pharmaceutical Sciences, MOE Key Laboratory of Bioorganic Phosphorus Chemistry & Chemical Biology, Tsinghua University, Beijing 100084, China; 3 Department of Thoracic Surgery, National Cancer Center/National Clinical Research Center for Cancer/Cancer Hospital, Chinese Academy of Medical Sciences and Peking Union Medical College, Beijing 100021, China; 4 Institute of Cancer Research, Henan Academy of Innovations in Medical Sciences, Zhengzhou, Henan Province 450000, China

**Keywords:** esophageal squamous cell carcinoma, NRF2, degraders, targeted therapy

## Abstract

NRF2 oncogenic activation (NRFA)-subtype esophageal squamous cell carcinoma (ESCC) patients exhibit poor prognosis and limited response to conventional therapies due to excessive NRF2 protein accumulation. Here, we identify L013, an NRF2 degrader, through high-content screening of 725 cereblon (CRBN) ligand-based degraders. Our results indicate that L013 effectively reduces NRF2 abundance and decreases *NFE2L2* mRNA expression in ESCC cells. Mechanistically, L013 promotes NRF2 ubiquitination through a ubiquitin-proteasome system (UPS)-associated process involving Cullin-dependent E3 ligase activity, while its A51-derived scaffold independently contributes to NRF2 suppression, at least in part, by impairing *NFE2L2* transcriptional output. In preclinical models, L013 reduces NRF2 levels and suppresses tumor growth in patient-derived organoids (PDOs) of ESCC and lung squamous cell carcinoma (LUSC), as well as ESCC xenograft models. No overt systemic toxicity occurs under the tested dosing regimen. Our findings establish L013 as a promising therapeutic candidate for NRFA-subtype ESCC patients, offering a potential strategy to target NRF2-driven oncogenesis.

## Highlights

• Chemically diverse screen identifies L013 as an NRF2-targeting compound
• L013 suppresses NRF2 in NRFA-subtype esophageal squamous cell carcinoma
• L013 integrates UPS-associated NRF2 degradation and A51-derived suppression of NRF2
• L013 displays balanced efficacy and safety in preclinical models

## Graphical abstract

**Figure:**
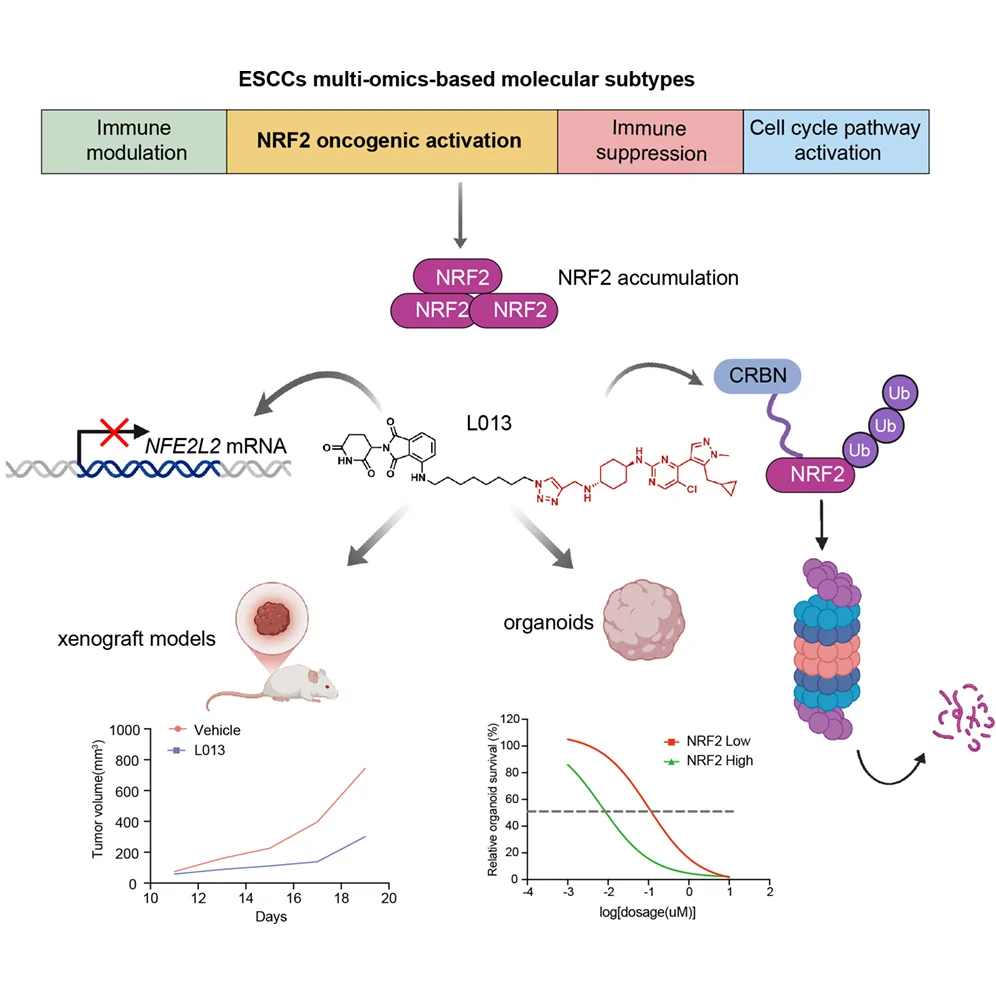

## Teaser

Yu et al. identify L013 as an NRF2-targeting agent that reduces NRF2 via UPS-associated degradation and A51-mediated suppression. L013 inhibits tumor growth in preclinical models, revealing therapeutic potential for NRFA-subtype ESCC.

## Introduction

Esophageal squamous cell carcinoma (ESCC) is a highly malignant tumor, ranking eleventh in incidence and seventh in mortality worldwide.^1^ The prognosis for ESCC patients remains poor, with a 5-year survival rate below 40%.^2^ Current treatments, including surgery, radiotherapy, chemotherapy, and immunotherapy, are limited in efficacy, highlighting the need for novel targeted therapies.^3^^,^^4^ Advances in multi-omics sequencing have improved our understanding of ESCC molecular subtyping,^5^^,^^6^ yet the discovery of new therapeutic targets remains limited. The development of precision therapies for ESCC patients is crucial to alleviating their disease burden.

We previously performed whole-genome sequencing on 508 ESCC samples and identified 10 missense mutations in the NRF2 protein-coding gene *NFE2L2* in 31 patients, corresponding to a mutation frequency of 6.1%. These mutations were significantly associated with poor prognosis in ESCC patients.^7^ We further analyzed paired samples from 155 ESCC patients using multi-omics sequencing and classified ESCC into four molecular subtypes: cell cycle signal activation (CCA), NRF2 oncogenic activation (NRFA, 24.5%, *n* = 38), immune suppression (IS), and immune modulation (IM).^8^ Among these, the NRFA subtype is particularly notable, as alterations in the NRF2 pathway drive its activation, leading to excessive NRF2 accumulation. Compared with the IS and IM subtype patients, NRFA patients exhibit low levels of immune infiltration, suggesting limited responsiveness to immunotherapy. Additionally, NRF2 overactivation in ESCC has been linked to resistance to both chemotherapy and radiotherapy,^9^^,^^10^ indicating that patients with this subtype may derive limited benefit from conventional treatment approaches. These findings underscore an urgent need for the development of novel targeted therapies for NRFA-subtype ESCC patients to improve clinical outcomes and enhance their quality of life.

NRF2 is a key transcription factor that maintains cellular redox homeostasis by regulating genes with antioxidant response elements (AREs) in their promoters. It is involved in critical cellular processes such as metabolism, oxidative stress response, autophagy, and apoptosis.^11^ Its unresolved crystal structure hinders the development of NRF2-targeted therapeutics. Physiologically, NRF2 is degraded by the KEAP1-CUL3 E3 ligase complex to maintain low intracellular levels; stress blocks this degradation, leading to nuclear NRF2 accumulation and downstream target gene activation.^12^ In cancer, mutations in *NFE2L2*, *KEAP1*, and *CUL3* disrupt this regulatory mechanism, leading to NRF2 overactivation. Aberrant NRF2 activity promotes tumor cell proliferation, resistance to apoptosis, angiogenesis, metabolic reprogramming, and metastasis.^13^ Although *NFE2L2* mutations were first identified in lung squamous cell carcinoma (LUSC), recent studies indicate that they occur most frequently in ESCC.^14^^,^^15^^,^^16^ Overall, activating mutations in *NFE2L2* are commonly observed in various squamous cell carcinomas (SCCs).

Tumor cells are often “addicted” to NRF2 overactivation, making NRF2 inhibition a potential strategy to impede cancer progression.^17^ However, the intrinsically disordered structure of NRF2 and the absence of a defined binding pocket for small-molecule inhibitors pose significant challenges to drug development. Currently, no specific NRF2 inhibitors are clinically available. ML385, the only small molecule reported to bind directly to NRF2, still requires further validation of its efficacy and specificity.^18^ Most reported NRF2 inhibitors are natural compounds with unclear mechanisms of action and limited specificity.^19^ Additionally, inhibitors and degraders targeting other components of the NRF2 signaling pathway are under development.^20^^,^^21^ Despite significant progress in elucidating the functional roles of NRF2, the development of therapeutic strategies targeting this pathway has not kept pace.

This study focused on achieving NRF2 degradation via targeted protein degradation (TPD)—a therapeutic strategy that harnesses the endogenous ubiquitin-proteasome system (UPS) to induce selective protein elimination. Conventional TPD modalities primarily include bifunctional proteolysis-targeting chimeras (PROTACs) and molecular glue degraders (MGDs), both of which facilitate target degradation through UPS-mediated proteolysis following ternary complex formation with E3 ubiquitin ligases. Despite sharing a common degradation pathway, these modalities differ fundamentally in their chemical architecture and mechanisms of target recognition. PROTACs are heterobifunctional compounds comprising a target-binding ligand and an E3 ligase-binding ligand connected by a chemical linker. In contrast, MGDs are monovalent molecules that enhance native target-E3 interactions through conformational remodeling, without direct dual binding.^22^^,^^23^^,^^24^ While PROTACs offer broad applicability through rational linker design, their relatively large molecular size imposes pharmacokinetic limitations. MGDs, on the other hand, exhibit more favorable drug-like properties but face challenges in rational design. Recent advances in understanding the structural principles governing MGD-target interactions may broaden their therapeutic potential.^25^^,^^26^^,^^27^ Notably, emerging degrader modalities, including intramolecular bivalent glues (IBGs), degradation tails, indirect degraders, nuclear hormone receptor degraders, SUMO-dependent degraders, and inhibitor-induced destabilizers, continuously redefine the TPD landscape.^28^ These innovative approaches not only transcend the classical PROTAC/MGD paradigm but also blur the traditional categorical boundaries, offering promising frameworks for developing next-generation therapeutic degraders.

By utilizing the ubiquitin-proteasome degradation pathway, TPD facilitates the degradation of specific target proteins. Transient or low-abundance ternary complexes are sufficient to trigger protein degradation. Unlike conventional inhibitors that require high-affinity binding to precisely engage their target, TPD can utilize ligands with lower affinity, promoting rapid assembly and disassembly of the ternary complex.^29^ This study proposes to exploit this lower-affinity characteristic of TPD to circumvent the need for precise structural targeting, thereby identifying degraders capable of inducing NRF2 degradation. Such an approach may offer an effective treatment strategy for patients with the NRFA subtype of ESCC, providing a promising avenue for targeted therapy.

## Results

### Elevated NRF2 expression is associated with poor prognosis in ESCC

In our previous research, we found that the NRFA subtype is an important molecular subtype of ESCC. This subtype is characterized by alterations in NRF2 pathway genes (*NFE2L2*, *CUL3*, and *KEAP1*) and by the upregulation of *NFE2L2*, which encodes NRF2, as well as *SOX2*.^8^ Further immunohistochemical analysis revealed that the protein level of NRF2 in the NRFA subtype was significantly higher than that in other subtypes, indicating that high NRF2 expression, particularly at the protein level, is a defining feature of a subset of ESCC patients.

In this study, we used immunohistochemistry (IHC) to assess NRF2 expression in ESCC patients and further evaluated its association with prognosis. In a cohort of 85 patients, high NRF2 expression was significantly associated with poor prognosis (*p* = 0.015) (Figures 1A and 1B). In another cohort of 286 ESCC patients, we similarly observed a significant association between high NRF2 expression and poor prognosis (*p* = 0.036) (Figure 1C**)**. Furthermore, analysis of The Cancer Genome Atlas (TCGA) ESCC dataset revealed that elevated *NFE2L2* mRNA expression might be a risk factor for poor prognosis, although the statistical difference did not reach significance, potentially due to discrepancies between mRNA and protein levels (Figures S1A and S1B). Collectively, these results indicate that elevated NRF2 expression, particularly at the protein level, is a key factor associated with poor prognosis in ESCC patients.

**Figure 1:**
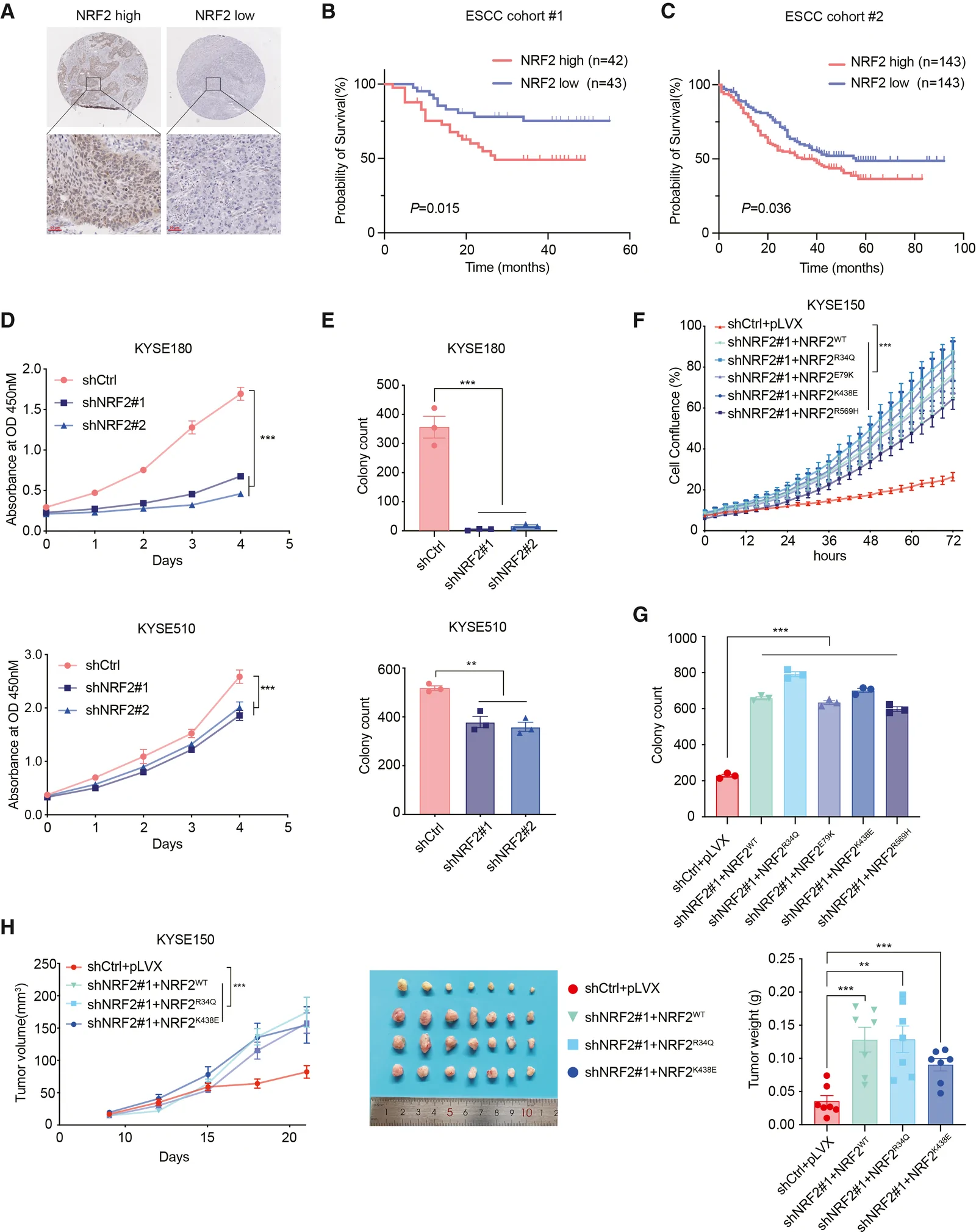
Aberrantly high NRF2 expression is associated with poor prognosis and malignant phenotypes in ESCC (A) Representative immunohistochemical images demonstrating high and low NRF2 expression in ESCC tissues. Scale bars represent 50 μm. (B) Kaplan-Meier survival curve for ESCC cohort #1 (*n* = 85), comparing survival probability between patients with NRF2 high expression (*n* = 42) and those with NRF2 low expression (*n* = 43). *p* values were determined by log rank test. (C) Kaplan-Meier survival analysis for ESCC cohort #2 (*n* = 286), with NRF2-high (*n* = 143) and NRF2-low (*n* = 143) expression groups. *p* values were determined by log rank test. (D) *In vitro* growth of KYSE180 and KYSE510 cells expressing control (shCtrl) or NRF2 shRNAs. (E) Colony formation assays of KYSE180 and KYSE510 cells expressing control (shCtrl) or NRF2 shRNAs. (F) *In vitro* proliferation curves of KYSE150 cells expressing shCtrl or NRF2 shRNA#1 followed by overexpression of wild-type and mutant *NFE2L2*. The growth curves of KYSE150 cells over 72 h were determined using the real-time, dynamic live-cell imaging system Incucyte S3. (G) Colony formation assays of KYSE150 cells expressing shCtrl or NRF2 shRNA#1 followed by overexpression of wild-type and mutant *NFE2L2*. (H) Representative images, tumor volume changes, and tumor weights of KYSE150 xenografts expressing shCtrl or NRF2 shRNA#1 followed by the overexpression of wild-type and mutant *NFE2L2*. *n* = 6 biological replicates in (D) and (F); *n* = 3 biological replicates in (E) and (G); and *n* = 7 biological replicates in (H). Data are represented as mean ± SD in (D) and (F) and as mean ± SEM in (E) and (G). ∗*p* < 0.05, ∗∗*p* < 0.01, and ∗∗∗*p* < 0.001 by Student’s *t* test in (E), (G), and (H) or by two-way ANOVA with Dunnett’s multiple-comparison test in (D), (F), and (H). See also Figure S1.

### NRF2 hyperactivation promotes ESCC cell proliferation and tumorigenesis

Building on the clinical observation that high NRF2 expression correlates with poor prognosis in ESCC, we next investigated its functional relevance in ESCC cell lines. We first assessed NRF2 expression levels in human ESCC cell lines and normal esophageal epithelial cells maintained in our laboratory. Notably, elevated NRF2 expression was observed not only in KYSE70 and KYSE180 cell lines harboring *NFE2L2* mutations (resulting in constitutive NRF2 activation) but also in the mutation-negative KYSE510 cell line (Figure S1C). These three cell lines have been previously documented in the literature as models of NRF2 hyperactivation.^15^ To investigate the functional role of NRF2, we performed NRF2 knockdown in KYSE180 and KYSE510 cells (Figure S1D). Subsequent CCK-8 and colony formation assays revealed that NRF2 depletion markedly suppressed cell proliferation and tumorigenic potential, indicating that the proliferative capacity of these cancer cells is dependent on aberrant NRF2 activation (Figures 1D and 1E; Figure S1E).

We next explored the effects of exogenous overexpression of wild-type (WT) and mutant NRF2 proteins on ESCC cell proliferation and tumorigenesis. On the basis of recurrent *NFE2L2* mutational sites identified through whole-genome sequencing of ESCC patients in our previous study and reported hotspots in the literature,^7^ we constructed plasmids harboring four high-frequency mutations for functional characterization. To eliminate potential interference from endogenous NRF2, KYSE150 cells were first subjected to short hairpin RNA (shRNA)-mediated knockdown targeting the 3′ UTR of endogenous *NFE2L2*, followed by ectopic expression of WT or mutant constructs. Knockdown efficiency and exogenous overexpression were validated by immunoblotting and qPCR using primers specific to the 5′ UTR and coding sequence (CDS) regions of *NFE2L2*, which allowed us to distinguish total versus endogenous NRF2 expression (Figures S1F and S1G). Cell proliferation and colony formation assays showed that overexpression of both WT and mutant NRF2 significantly promoted the *in vitro* ESCC cell proliferation and tumorigenicity (Figures 1F and 1G; Figure S1H). Additionally, subcutaneous xenograft models in nude mice confirmed that exogenous NRF2 expression enhanced tumor growth *in vivo*, with significantly increased tumor volume and weight compared with the control group (Figure 1H). Taken together, these results demonstrate that NRF2 hyperactivation drives the proliferative and tumorigenic capacities of ESCC cells.

### High-content screening identifies L013 as a selective NRF2 degrader

Given the aberrantly high NRF2 expression in the NRFA subtype and its association with poor prognosis and malignant phenotypes in ESCC, we aimed to identify targeted degraders of NRF2 as a potential therapeutic strategy for patients with this subtype. We performed the high-content screening using a library containing 725 cereblon (CRBN) ligand-based degraders. To ensure extensive coverage of the chemical space, a scaffold-centric diversity strategy was adopted in the design of this library. Ligands representing distinct chemical scaffolds, including a diverse range of heterocyclic backbones, were selected for library construction. This structural heterogeneity endows the degraders with the capacity to explore orthogonal binding modes and engage with distinct subpockets within the target protein, thereby maximizing the probability of identifying viable conformations of the ternary complex.

We established a stable KYSE150 cell line overexpressing a green fluorescent protein (GFP)-NRF2 fusion protein for screening a degrader library. GFP served as a reporter for NRF2 degradation: degrader-induced degradation of the NRF2 fusion protein would result in a corresponding loss of green fluorescence (Figure 2A). When NRF2 protein was degraded by a degrader molecule, intact GFP fluorescence in cells appeared as scattered green fluorescent particles. Therefore, the number of these particles was used as a measure of NRF2 protein degradation. Using this criterion, we identified the top 100 degrader candidates with potential NRF2-degrading activity (Figure 2B**)**. To exclude false-positive results caused by poor compound solubility, we eliminated degraders such as N025 and RP023, which precipitated in the culture medium (Figure S2A). Finally, three degrader molecules, L013, N024, and N026, were selected for subsequent evaluation of their NRF2-degrading potential (Figure 2C**)**.

**Figure 2:**
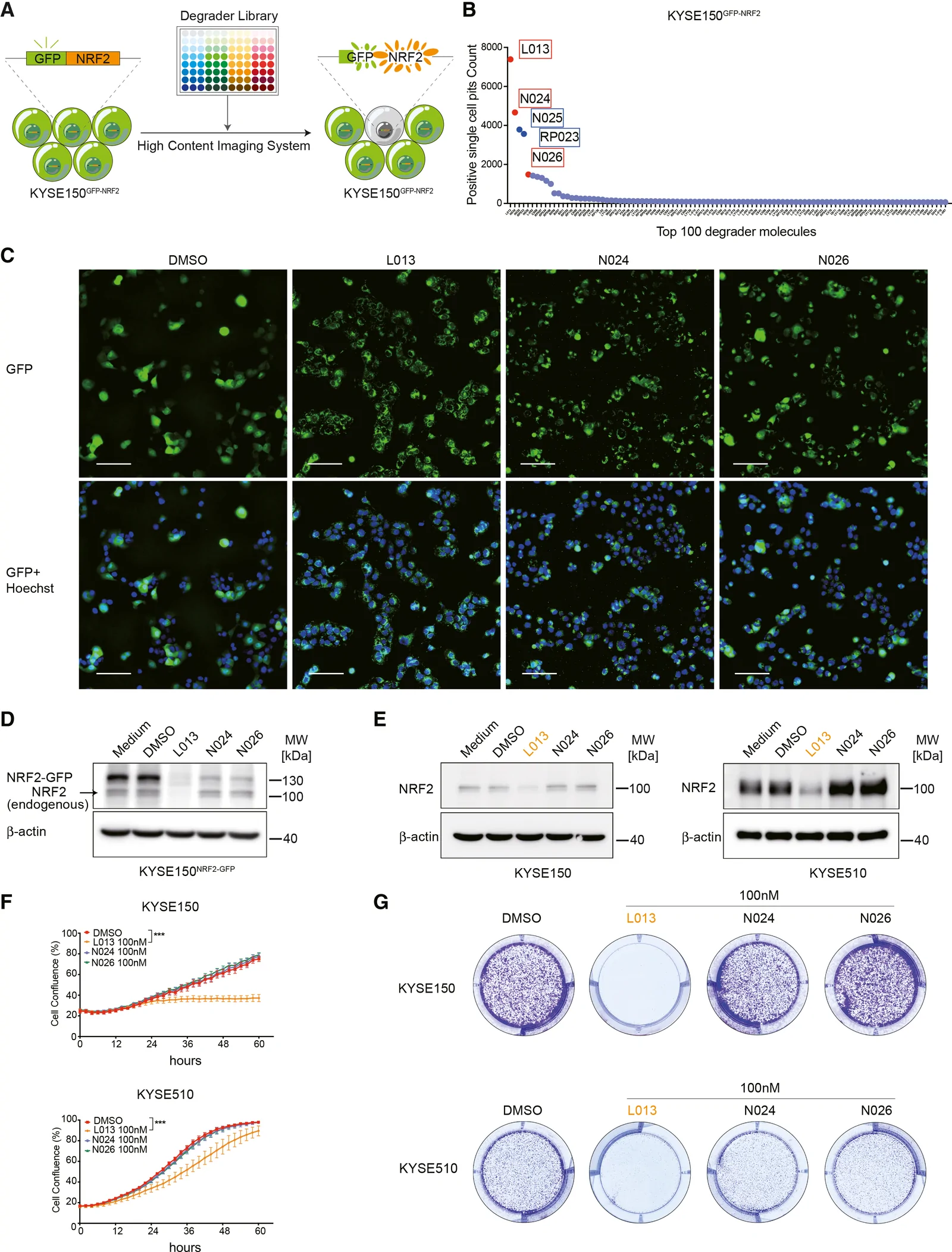
High-content screening identifies L013 as a selective NRF2 degrader (A) Schematic diagram of degrader molecule screening for NRF2 degradation, using a high-content system. Upon degradation of the GFP-NRF2 fusion protein by the degrader molecules in the library, the green fluorescence of GFP is quenched, resulting in a shift of the fluorescence from a continuous sheet-like pattern to punctate granules. (B) Top 100 degraders based on the analysis of green fluorescent particle counts in positive cells, using a high-content imaging system. Degradation of the GFP-NRF2 fusion protein causes the green fluorescence to change from a continuous sheet-like pattern to punctate granules. Using a customized high-content analysis pipeline, we considered the number of scattered green punctate fluorescent granules within cells as the primary readout to evaluate the degradation activity of degrader molecules. (C) Representative images captured using the high-content imaging system after the addition of indicated degraders at 5 μM for 12 h. GFP represents the green fluorescence of the entire cell, while Hoechst indicates the position of the cell nucleus. Scale bars represent 100 μm. (D) Immunoblotting to detect the levels of exogenous NRF2-GFP fusion protein and endogenous NRF2 protein in KYSE150 cells stably overexpressing NRF2-GFP fusion protein after treatment with 5 μM degraders for 12 h. (E) Immunoblotting to detect the levels of endogenous NRF2 protein in KYSE150 and KYSE510 cell lines after treatment with 5 μM degraders for 12 h. (F) Cell proliferation curves for KYSE150 and KYSE510 cell lines after treatment with degraders. *n* = 5 biological replicates. Data are represented as mean ± SD. ∗∗∗*p* < 0.001 by two-way ANOVA with Tukey’s multiple-comparison test. (G) Representative images showing the *in vitro* colony formation of KYSE150 and KYSE510 cell lines after treatment with degraders for 7 days. See also Figure S2.

We first treated KYSE150 cells stably overexpressing the NRF2-GFP fusion protein—which were used in the high-content screening—with the three selected degraders—and measured both exogenous fusion protein levels and endogenous NRF2 levels. We found that, although all three degrader molecules could degrade the exogenously overexpressed fusion protein, only L013 was able to degrade the endogenous NRF2 protein (Figure 2D**)**. Treatment of ESCC cell lines with the three degraders yielded results consistent with those observed in the exogenously overexpressed cells. Specifically, only L013 exhibited a degrading effect on endogenous NRF2 protein (Figure 2E; Figure S2B). Additionally, we employed the drug affinity responsive target stability (DARTS) assay to examine the binding interactions between the degraders and NRF2 protein. Using BSA as a control, we found that L013 provided significant protection to the NRF2 protein, indicating the binding between them. Compared with the small-molecule inhibitor ML385, which has been reported to bind to NRF2, L013 showed a stronger binding affinity for the NRF2 protein. Meanwhile, NRF2 protein also exhibited a weaker binding to N024 (Figure S2C). *In vitro* proliferation and colony formation assays in ESCC cell lines further demonstrated that L013 exerted significant inhibitory effect on ESCC cells, whereas N024 and N026 did not show significant effects (Figures 2F and 2G; Figures S2D and S2E). Altogether, these results identify L013 as a potent NRF2 degrader discovered through high-content screening.

### L013 exhibits rapid and selective NRF2 degradation

Structurally, L013 consists of three components: a ligand for binding the target protein, a ligand for recruiting the E3 ligase CRBN, and a linker that anchors the target protein to the E3 ubiquitin ligase (Figure 3A). In order to characterize L013, we conducted time-dependent degradation experiments using ESCC cell lines. Upon treatment with L013, rapid NRF2 protein degradation was observed, resulting in approximately 50% reduction within 4 h (Figure 3B**)**. To further determine the efficacy of L013 in degrading NRF2, we performed a dose-dependent degradation assay in ESCC cell lines. After 8 h of treatment, nearly complete NRF2 degradation was observed in all tested cell lines at a concentration of 500 nM. (Figure 3C; Figure S3A). In addition, L013 eliminated the proliferative advantage conferred by NRF2 overexpression, effectively suppressing the *in vitro* proliferation of NRF2-overexpressing cells to a level comparable to that of control cells (Figure 3D; Figure S3B). Furthermore, in NRF2-knockout cells, L013 no longer exerted a significant anti-proliferative effect compared with DMSO treatment (Figure 3E; Figure S3C). To further evaluate the degradation selectivity of L013, we examined the degradation of other NRF2 family proteins, such as NRF1 and NRF3, at different time points. We found that L013 selectively reduced both the protein and mRNA levels of NRF2 without affecting other members of the same family. (Figure 3F; Figure S3D). These results indicate that L013 is a potent and selective degrader of NRF2. To further confirm the specificity of L013-mediated inhibition of NRF2, we examined the protein levels of its upstream regulators CUL3 and KEAP1 after L013 treatment. The results showed that the protein levels of both CUL3 and KEAP1 remained unchanged. (Figure 3G**)**. Together, these findings establish L013 as a rapid and selective NRF2 degrader, underscoring its potential as a promising agent for NRF2-targeted therapies.

**Figure 3:**
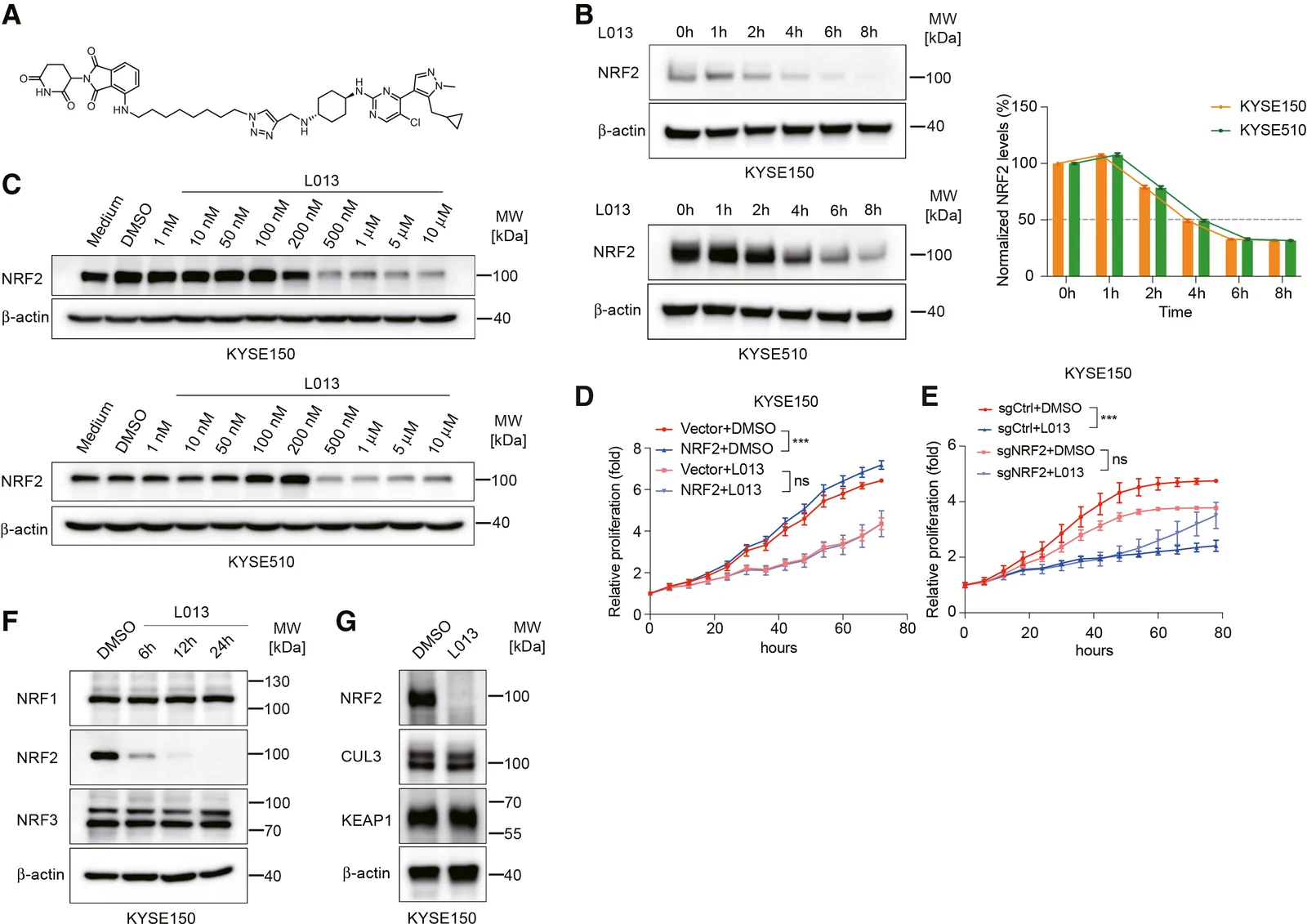
L013 exhibits rapid and selective NRF2 degradation (A) Structure of L013. (B) Immunoblotting was used to detect NRF2 degradation by 1 μM L013 over the indicated time in KYSE150 and KYSE510 cells (left). Quantification was done via grayscale analysis, with ratios normalized to the 0 h group (right). (C) Immunoblotting was used to detect NRF2 degradation by L013 under the indicated concentration for 8 h in the KYSE150 and KYSE510 cell lines. (D) Cell proliferation curves for KYSE150 cells expressing vector or NRF2 and treated with or without 100 nM L013. (E) Cell proliferation curves for KYSE150 cells expressing non-target sgRNA(sgCtrl) or NRF2 sgRNA and treated with or without 100 nM L013. *n* = 6 biological replicates. (F) Immunoblotting was used to detect the protein levels of NRF1, NRF2, and NRF3 in KYSE150 cells treated with L013 at 1 μM over the indicated time. (G) Immunoblotting was used to detect the protein levels of NRF2, CUL3, and KEAP1 in KYSE150 cells treated with L013 at 1 μM for 6 h. Data are represented as the mean ± SD in (D) and (E). ∗∗∗*p* < 0.001, by two-way ANOVA with Tukey’s multiple-comparison test in (D) and (E). See also Figure S3.

### L013 promotes UPS-associated NRF2 reduction, while retaining an A51-derived NRF2-suppressive activity

To define how L013 lowers NRF2, we first examined NRF2 ubiquitination after L013 treatment. L013 markedly increased NRF2 ubiquitination, supporting involvement of the UPS in NRF2 reduction (Figure 4A; Figure S4A). We next examined NRF2 ubiquitination following treatment with MLN4924, a highly selective inhibitor of the NEDD8-activating enzyme.^30^ MLN4924 inhibits the activity of Cullin RING ligases, including CRBN-containing CRL4 complexes and CUL3-based E3 ligases. Combined treatment with MLN4924 and L013 did not further increase NRF2 ubiquitination (Figure 4B**)**, suggesting that the increase in NRF2 ubiquitination induced by L013 involves a Cullin-dependent E3 ligase activity and is consistent with the participation of CRBN-associated complexes. We further exposed cells overexpressing Flag-tagged CRBN to L013 for a short period and found that L013 enhanced the interaction between NRF2 and CRBN (Figure 4C**)**. Collectively, these findings support a model in which L013 promotes NRF2 degradation, at least in part, through a Cullin-dependent, CRBN-associated ubiquitination process. However, MLN4924 only partially reversed the reduction in NRF2 protein levels induced by L013 (Figure 4D**)**. Similarly, the proteasome inhibitor MG132 only partially restored NRF2 abundance following L013 treatment (Figure 4E**)**. These observations indicate that degradation through the ubiquitin-proteasome pathway accounts for only part of the inhibitory effect of L013 on NRF2, suggesting that additional mechanisms are also involved. L013 was unexpectedly identified from a screen of a chemically diverse compound library and contains an A51-derived scaffold (Figure 4F**)**. Because A51 has been reported as a CK1α inhibitor, we next examined whether the A51-derived moiety also contributes to NRF2 suppression.^31^ Notably, both L013 and A51 rapidly reduced NRF2 protein abundance and decreased *NFE2L2* mRNA levels (Figures 4G and 4H; Figures S4C and S4D). These findings reveal a previously unappreciated NRF2-lowering activity associated with the A51 scaffold and suggest that the activity of L013 cannot be attributed solely to classical degrader-mediated ubiquitination. The A51 scaffold appears to contribute to NRF2 suppression, at least, at the transcriptional level. Whether it also promotes NRF2 degradation through a molecular glue-like mechanism remains unresolved. We next compared NRF2 engagement by L013 and A51, using DARTS and surface plasmon resonance (SPR). In both assays, L013 showed stronger apparent interaction with NRF2 than A51 (Figures 4I and 4J; Figures S4E and S4F). Isothermal titration calorimetry (ITC) and microscale thermophoresis (MST) further confirmed micromolar binding of L013 to recombinant full-length NRF2 (Figures 4K and 4L**)**. Together, these data indicate that L013 retains the ability to recognize NRF2 and has the potential to exhibit favorable overall performance in subsequent preclinical efficacy and safety evaluations.

**Figure 4:**
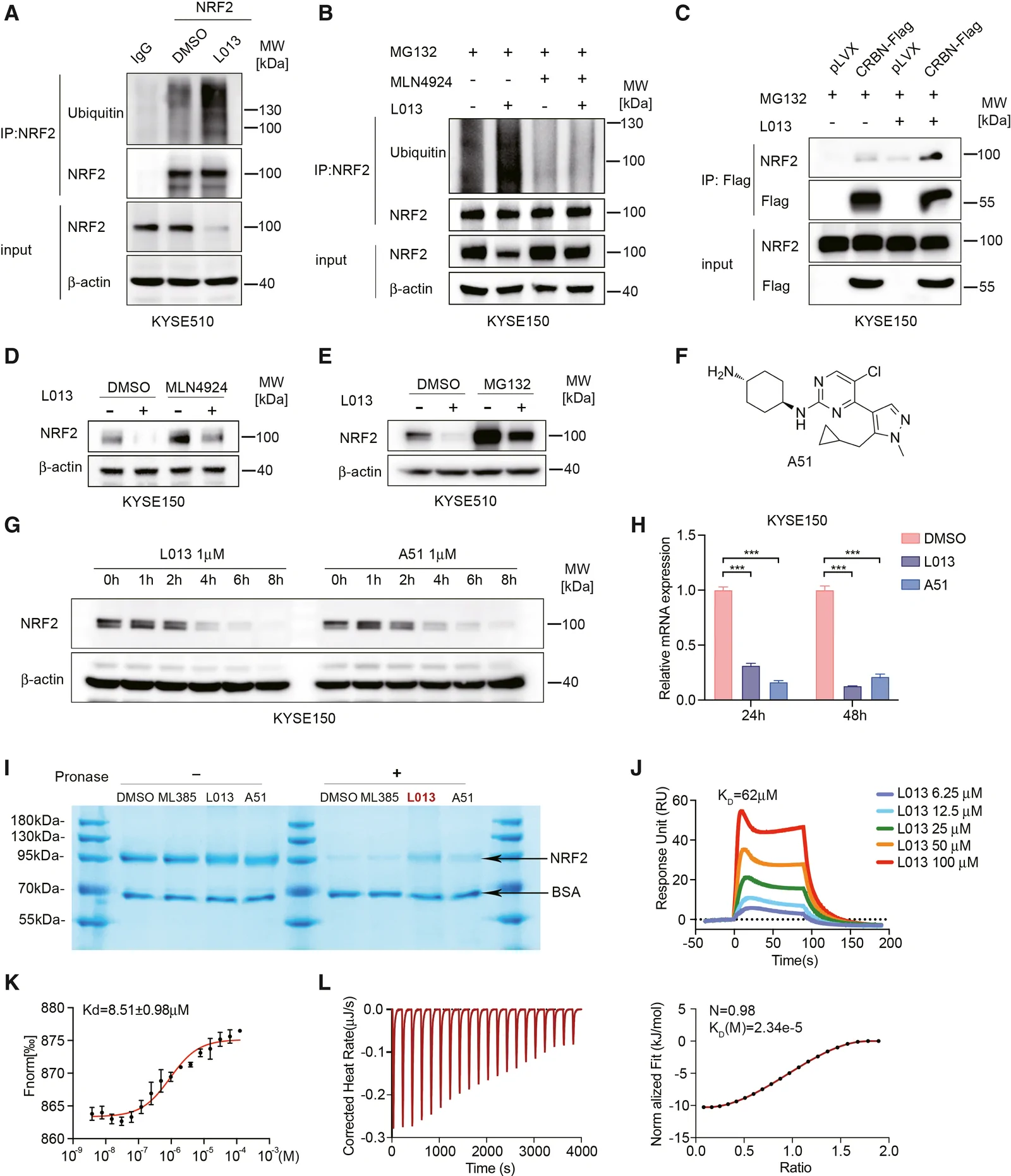
L013 promotes UPS-associated NRF2 reduction, while retaining an A51-derived NRF2-suppressive activity (A) Immunoblotting was used to detect NRF2 ubiquitination in KYSE510 cells pretreated with 10 μM MG132 for 4 h followed by treatment with or without 1 μM L013 for an additional 4 h. IgG was used as negative control. (B) Immunoblotting detection for NRF2 ubiquitination in KYSE150 cells. The cells were pretreated with 10 μM MG132 for 4 h, then incubated for another 4 h with 1 μM L013, 1 μM MLN4924, or the combination of L013 and MLN4924. (C) Anti-Flag co-immunoprecipitation was performed to assess the association between CRBN-Flag and endogenous NRF2. Cells overexpressing vector or CRBN-Flag were pretreated with 10 μM MG132 for 2 h, and then incubated with 500 nM L013 for 1 h. (D) Immunoblotting detection for NRF2 in KYSE150 cells. The cells were pretreated with 500 nM MLN4924 for 4 h, followed by 500 nM L013 for 24 h. (E) Immunoblotting detection for NRF2 after a 2-h pretreatment with MG132 (2 μM), followed by a 12-h treatment with L013 (1 μM) in KYSE510 cells. (F) Chemical structure of A51. (G) Immunoblotting detection for NRF2 degradation at the indicated time points after addition of L013 or A51 in KYSE150 cells. (H) qPCR analysis of NRF2 expression levels in KYSE150 cells at the indicated time after the addition of 1 μM L013 or A51. *n* = 3 biological replicates; data are presented as the mean ± SEM. ∗∗∗*p* < 0.001, by Student’s *t* test. (I) Upon addition of pronase, L013-treated NRF2 showed an obvious higher band intensity compared with the other groups, while the BSA band intensity was comparable between the groups. (J) Biacore kinetic analysis demonstrating the affinity of L013 for full-length NRF2 protein. (K) ITC assay between L013 and NRF2 full-length protein. (L) MST analysis of L013 binding to recombinant NRF2 full-length protein. See also Figure S4.

To further investigate the reduction in *NFE2L2* mRNA, cells were co-treated with actinomycin D and L013. Under transcriptional blockade, L013 did not further accelerate the decline in *NFE2L2* mRNA levels (Figure S4G), arguing against a major effect on mRNA stability and supporting a transcriptional mechanism. Given prior reports that A51 inhibits RNA Pol II C-terminal domain (CTD) phosphorylation at Ser2 and Ser5 (pSer2/5 CTD), we analyzed pSer2 CTD chromatin immunoprecipitation sequencing (ChIP-seq) data from A51-treated cells at the murine *Nfe2l2* locus.^31^ Corresponding regions in the human *NFE2L2* gene (transcription start site, TSS; gene body) were examined using qPCR primers. The results showed that L013 reduced pSer2 CTD occupancy across the *NFE2L2* region in ESCC cells and also decreased RNA Pol II CTD phosphorylation at Ser2 (Figures S4H and S4I), consistent with impaired transcriptional elongation contributing to reduced *NFE2L2* mRNA abundance. However, these data do not, by themselves, establish direct promoter-specific repression of *NFE2L2* transcription. Collectively, our results support a model in which L013 combines a UPS-associated NRF2 degradation component with an A51-derived scaffold that independently contributes to NRF2 suppression.

### L013 suppresses tumor growth in NRF2-hyperactivated organoids and xenografts

ESCC is a common subtype of SCCs. Integrated genomic, epigenetic, and transcriptomic analyses have revealed shared genomic alterations across different SCC types, with activating mutations in *NFE2L2* reported to occur recurrently.^15^^,^^32^ Given these conserved molecular features, therapeutic strategies that target SCCs as a unified entity may hold promise. To test this hypothesis, we established patient-derived organoids (PDOs) from three ESCC and two LUSC cases. These organoids were treated with varying concentrations of L013, and viability assays were performed 72 h post-treatment along with morphological assessments. Notably, 1 μM L013 significantly suppressed viability across all organoids, while 100 nM L013 selectively inhibited ESCC#3 and LUSC#1 organoids (Figures 5A and 5C). Drug sensitivity profiling based on half-maximal inhibitory concentration (IC_50_) values demonstrated markedly lower IC_50_ values in ESCC#3 and LUSC#1 than in other organoids (Figures 5B and 5D**)**. IHC analysis further revealed elevated NRF2 expression in ESCC#3 and LUSC#1, correlating with their heightened sensitivity to L013 (Figures 5E and 5F; Figures S5A and S5B). To further explore the biological basis underlying the heterogeneous sensitivity of PDOs to L013, we performed RNA sequencing (RNA-seq) and analyzed the expression of NRF2 pathway genes. Notably, the most sensitive PDOs (ESCC#3 and LUSC#1) displayed significantly higher expression of *NFE2L2* and most of its downstream pathway-related genes at the transcriptional level, suggesting that NRF2 pathway activation is associated with enhanced responsiveness to L013 (Figures S5C and S5D). These findings demonstrate heightened responsiveness to L013 in NRF2-hyperactivated organoid models, providing preliminary evidence suggesting potential activity of L013 in NRF2-activated SCC models beyond ESCC. We further examined the degradation of NRF2 in response to L013 treatment in ESCC and LUSC PDOs, and the results showed that L013 significantly degraded NRF2 in both PDOs (Figures 5G and 5H**)**.

**Figure 5:**
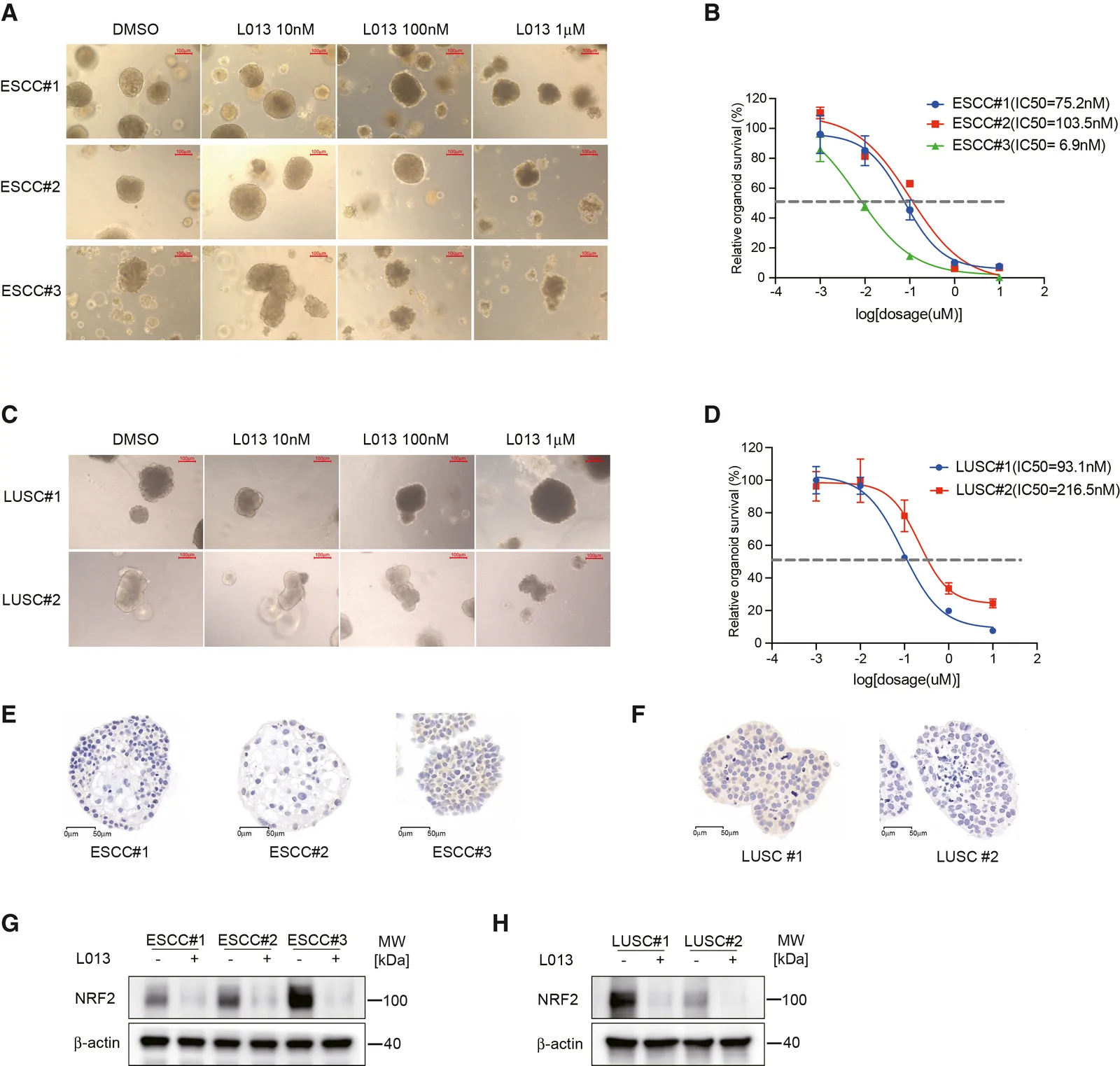
L013 suppresses viability of NRF2-hyperactivated organoids (A) Cell morphology assessment of ESCC organoids treated with increasing concentrations of L013 for 72 h. Scale bars represent 100 μm. (B) Dose-response curves of ESCC organoids after treatment with L013. IC_50_ values are indicated in parentheses. (C) Cell morphology assessment of LUSC organoids treated with increasing concentrations of L013 for 72 h. Scale bars represent 100 μm. (D) Dose-response curves of LUSC organoids after treatment with L013. IC_50_ values are indicated in parentheses. (E) IHC staining for NRF2 in ESCC organoids. Scale bars represent 50 μm. (F) IHC staining for NRF2 in LUSC organoids. Scale bars represent 50 μm. (G and H) Immunoblotting detection for NRF2 after 1 μM L013 treatment for 48 h in indicated PDOs. See also Figure S5.

To further evaluate the *in vivo* efficacy and safety of L013, we first performed pharmacokinetic studies of L013 in BALB/c nude mice via intravenous injection (i.v.) and oral administration (p.o.). The results showed that, despite low oral bioavailability, repeated oral administration produced a detectable pharmacodynamic effect in tumors, as reflected by reduced NRF2 levels (Figure S6). We then established subcutaneous xenograft models with oral administration of L013. L013 treatment significantly inhibited tumor growth, with the treated groups showing markedly reduced tumor volume and weight compared with the control groups (Figures 6A–6C). At a dose of 10 mg/kg, L013 induced no more than 10% body weight loss in mice, indicating acceptable systemic tolerance (Figure 6D**)**. Moreover, IHC staining revealed reduced NRF2 and Ki67 levels in the L013-treated group, consistent with the observed decrease in proliferative activity (Figure 6E**)**. Serum biochemical analyses showed no significant changes in liver or kidney function parameters between the L013-treated and control groups (Figures 6F–6H**)**. Furthermore, hematoxylin and eosin (H&E) staining of major organs revealed no histopathological abnormalities in either group, which indicates that L013 exhibits no overt systemic toxicity under the tested short-term dosing regimen (Figure 6I**)**. All these results demonstrate the favorable *in vivo* efficacy of L013.

**Figure 6:**
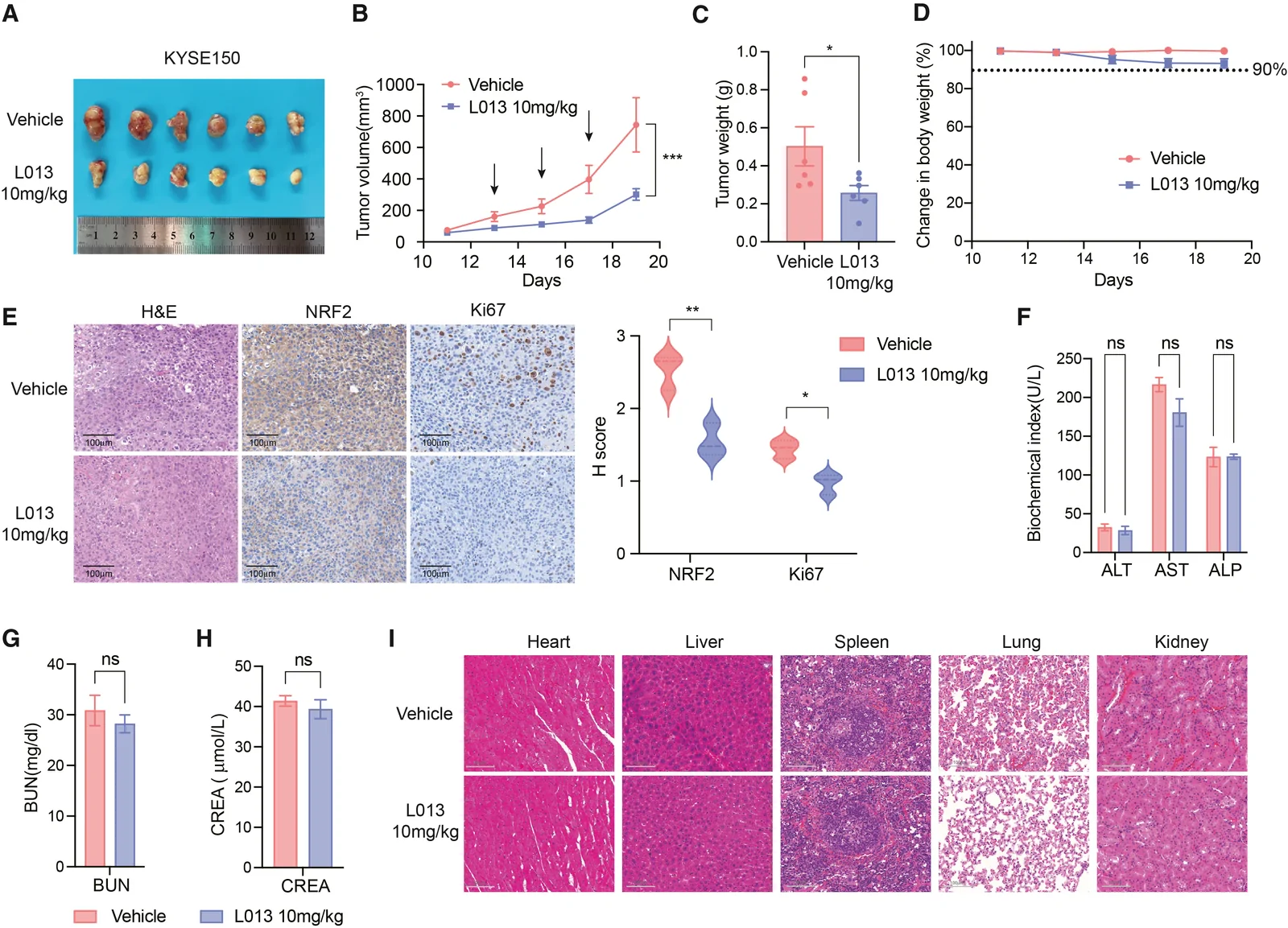
L013 effectively inhibits tumor growth in xenograft models (A) Representative images of KYSE150 xenografts treated with vehicle or L013 (10 mg/kg). (B) Tumor volume changes in KYSE150 xenografts treated with vehicle or L013 over time. (C) Tumor weight changes in KYSE150 xenografts treated with vehicle or L013. (D) Body weight changes in the mice bearing KYSE150 xenografts treated with vehicle or L013 over time. Dotted line represents the 90% change level. (E) Representative images and H-score of H&E staining and IHC staining for NRF2 and Ki-67 expression in the mouse xenografts. Scale bars represent 100 μm. *n* = 3 technical replicates from 6 biological replicates. (F–H) Biochemical indices in serum samples collected from vehicle- or L013-treated mice. (I) H&E staining of major organs from mice bearing KYSE150 xenografts treated with vehicle or L013 (10 mg/kg). Scale bars represent 100 μm. *n* = 6 biological replicates (B–D). *n* = 4 technical replicates from 6 biological replicates (F–H). Data are represented as mean ± SEM. ∗*p* < 0.05, ∗∗*p* < 0.01, and ∗∗∗*p* < 0.001, by two-way ANOVA with Šídák’s multiple-comparison test (B) or by Student’s *t* test (C, E, and F–H). See also Figure S6.

## Discussion

In cancer cells, constitutive NRF2 activation driven by pathway alterations confers survival advantages by upregulating antioxidant and detoxifying enzymes and reprogramming metabolism, thereby promoting tumor proliferation, apoptosis resistance, angiogenesis, and metastasis.^33^^,^^34^^,^^35^ To address the therapeutic challenge of targeting the NRFA subtype in ESCC, we developed L013, a targeted degrader of NRF2. The degradation efficacy of L013 was validated in cellular, animal, and organoid models. While our results show that L013 lowers the NRF2 levels and reduces *NFE2L2* mRNA abundance, we cannot exclude the possibility that the A51-derived segment of L013 promotes NRF2 degradation through a molecular glue-like mechanism. Collectively, L013 represents a promising targeted therapy for NRFA-subtype ESCC patients, offering alternative therapeutic options for this molecularly defined group.

TPD offers distinct advantages in cancer therapy, including improved selectivity, potency, and the potential to overcome drug resistance. As TPD continues to gain broader application, novel degrader modalities have emerged, underscoring the diversity and flexibility of this therapeutic strategy.^28^ In this study, the A51-derived scaffold of L013 was found to contribute to NRF2 suppression, at least partly, by reducing the *NFE2L2* transcriptional output. While our data demonstrate that L013 reduces NRF2 protein levels and *NFE2L2* mRNA abundance, the precise biochemical mechanism underlying these effects remains to be fully elucidated. In particular, the possibility that the A51-derived moiety contributes to NRF2 degradation through a molecular glue-like mechanism cannot be excluded based on current data. The dissociation constants (Kd) for the binding of L013 to NRF2 were determined to be 62, 23.4, and 8.51 ± 0.98 μM by SPR, ITC, and MST assays, respectively, with notable discrepancies among the results from different techniques. As an intrinsically disordered protein (IDP), NRF2 makes this finding a direct reflection of the inherent technical limitations of traditional biophysical approaches in characterizing IDP-ligand interactions. These techniques cannot avoid the detection biases stemming from the core properties of IDPs, namely the lack of a fixed three-dimensional structure and high conformational dynamism.^36^ Moreover, the prevalent weak binding feature between IDPs and their ligands further amplifies the systematic errors of various detection techniques, which precludes the acquisition of absolute and consistent affinity parameters and allows the measurement of only the apparent binding affinity. This represents a common technical challenge in investigating IDP interactions using conventional biophysical methods including SPR, ITC, and MST. Future studies should further elucidate the molecular mechanisms and structural determinants governing the interaction between L013 and NRF2. NRF2 has long been considered an “undruggable” target due to its intrinsically disordered structure.^17^ Limited structural data are available for NRF2, complicating the rational design of targeting strategies. However, recent advances in artificial intelligence-driven approaches for targeting IDPs may provide a framework for rational development of degraders against NRF2 and other IDPs.^37^ These innovations highlight the evolving landscape of TPD and its potential for addressing previously intractable therapeutic challenges. Notably, protein degraders such as PROTACs often possess large molecular weights (>800 Da) and high polarity, far exceeding the oral absorption limits defined by Lipinski’s Rule of Five. Previous studies have suggested poor membrane permeability and unclear uptake mechanisms for these macromolecules, but recent work has identified CD36 as a key transporter mediating the transmembrane transport of large-molecule drugs like PROTACs. Structural modifications to enhance CD36 binding have been shown to potentiate PROTAC activity, offering promise for the clinical translation of TPD.^38^ Clinically, the oral PROTAC estrogen receptor (ER) degrader ARV-471 has demonstrated encouraging efficacy in phase 3 trials for advanced breast cancer, highlighting the substantial potential of TPD in oncology.^39^ Together, these advancements bridge mechanistic insights with translational applications, positioning TPD as a transformative paradigm in precision cancer therapy.

As a master transcription factor, NRF2 orchestrates a broad array of downstream target genes.^40^ Therefore, degradation of NRF2 by L013 is expected to disrupt multiple signaling cascades, underscoring the critical need for patient stratification in clinical settings to minimize potential off-target effects. Previous studies from our group have identified a distinct molecular subtype of ESCC characterized by NRF2 oncogenic activation and elevated NRF2 expression. Functional validation in cell lines and PDOs confirmed heightened sensitivity to L013 in this subgroup, suggesting that the NRF2 activation status may serve as a predictive biomarker for therapeutic response. These findings highlight the potential of L013 as a precision therapy for ESCC patients with NRF2-driven oncogenesis, emphasizing the translational value of molecular stratification in guiding targeted interventions. Moreover, the commonalities in epidemiology, genomics, epigenetics, and immunobiology among SCCs support the development of unified therapeutic strategies across anatomical sites.^4^^,^^32^ The strong efficacy of L013 observed in LUSC organoids further suggests its potential to inhibit NRF2 activity in other SCC subtypes.

Furthermore, during tumorigenesis and progression, NRF2 has long been recognized as playing a dual role in both tumor promotion and suppression. This dichotomous function of NRF2 is supported by extensive experimental evidence. Most studies have suggested that NRF2 activation can prevent the transformation of normal cells into cancer cells through its antioxidant protective effects; however, sustained, constitutive NRF2 activity favors the survival of already transformed cancer cells.^13^ Given this dual role, a critical issue in applying NRF2-targeted therapies is determining the therapeutic window for NRF2, namely identifying which patients are suitable candidates for NRF2 inhibitors.^11^ Phylogenetic analysis has indicated that mutations in *NFE2L2*, the gene encoding NRF2, are typically located on the branches of tumor phylogenetic trees, suggesting that such mutations may represent relatively late events in ESCC development.^41^ This also implies that *NFE2L2* mutations could serve as biomarkers to guide the clinical application of NRF2 inhibitors. In our previous study, whole-genome sequencing of 508 ESCC paired samples revealed 10 *NFE2L2* missense mutations in 31 cases (6.1% frequency), with a striking association between these mutations and poor prognosis.^7^ The most frequent *NFE2L2* mutations occur within the domain mediating NRF2-KEAP1 interactions, leading to constitutive NRF2 activation.^12^ Identifying degraders that specifically target mutant NRF2 proteins in future studies may enable more precise treatment of patients with NRF2 oncogenic activation, reduce disruption of normal NRF2 functions, minimize adverse effects, and ultimately improve outcomes for ESCC patients. NRF2-hyperactive SCCs, including ESCC, have been reported to display an immune-evasive or “immune-cold” phenotype characterized by decreased immune cell infiltration.^8^^,^^42^ In ESCC, NRF2/NQO1 expression is also positively associated with PD-L1 expression, supporting a link between NRF2 pathway activation and immune checkpoint regulation.^43^ Mechanistically, NRF2 can restrain innate immune sensing and interferon output, whereas NRF2 loss or silencing enhances type I interferon/STING-related responses in multiple systems.^44^^,^^45^ These findings suggest that NRF2-targeted agents such as L013 may not only suppress tumor cell growth but may also help recondition an NRF2-driven immune-cold microenvironment. Although the present study did not test immunotherapy combinations, future work in immunocompetent models should determine whether L013 alters PD-L1 expression, interferon-response genes, and antigen-presentation programs, and whether these changes enhance responsiveness to PD-1/PD-L1 blockade or STING-directed immunotherapy.

In conclusion, through molecular subtyping of ESCC, we identified that patients with the NRFA subtype are characterized by excessive accumulation of NRF2 protein. Targeting this hallmark, we developed an NRF2-targeted degrader as a precision therapeutic strategy for NRFA cases. By screening a degrader library, we identified L013 as a potent degrader capable of efficiently degrading NRF2. Mechanistically, L013 effectively reduces NRF2 levels, at least in part, by promoting its ubiquitination-mediated degradation. Our current data also suggest the involvement of the A51-derived moiety, which may contribute to NRF2 reduction through a potential molecular glue-like mechanism. Furthermore, studies in mouse and organoid models confirmed the preclinical efficacy of L013 in targeting NRF2. Collectively, these findings establish L013 as a promising precision therapy for NRFA-subtype ESCC patients, with potential to improve survival outcomes in this molecularly defined population.

### Limitations of the study

While we identified L013 as a degrader of NRF2 and a potential precision-therapy strategy for ESCC patients with the NRFA subtype, the specificity and accuracy of L013-mediated NRF2 degradation remain to be fully clarified. Future studies are needed to fully elucidate the molecular mechanisms by which L013 mediates the degradation of NRF2. Moreover, given the complex roles of NRF2 within tumors and the tumor microenvironment, additional studies are needed to address the challenges of achieving precise local delivery of L013 in tumors. Finally, further preclinical studies are warranted to clarify the NRF2-degrading activity of L013 in ESCC as well as other SCCs and to determine its consequent antitumor effects.

## Resource availability

### Lead contact

Requests for further information, resources, and reagents should be directed to and will be fulfilled by the lead contact, Zhihua Liu (liuzh@cicams.ac.cn).

### Materials availability

This study generated the small molecule L013. Materials will be made available by the corresponding author(s) upon reasonable request and subject to applicable institutional regulations and material(s) transfer agreements.

### Data and code availability

• The RNA-seq data have been deposited in the Gene Expression Omnibus (GEO) database and are publicly available under accession number GEO: GSE324866.
• This paper does not report original code.
• Any additional information required to reanalyze the data reported in this work paper is available from the lead contact upon request.

## Acknowledgments

This research was funded by the Innovative Drug Research and Development—10.13039/501100018537National Science and Technology Major Project (2025ZD1800400), the 10.13039/501100001809National Natural Science Foundation of China (82188102, 82403741, 82330115, and 82125034), the 10.13039/501100012166National Key R&D Program of China (2021YFA1300200), the National High Level Hospital Clinical Research Funding (2025-LYZX-D-B03), and the Beijing Outstanding Young Scientist Program (JWZQ20240101007).

## Author contributions

Conceptualization, Z.L., Y.R., and Y.Z.; investigation, X.Y., Y. Wu, and X.S.; organoid establishment, R.H.; compound synthesis and structural characterization, Y.C., W.Z., and X.S.; formal analysis: X.Y., Y. Wu, and X.S.; animal studies, X.Y., Y.L., and Y. Wang; writing – original draft, X.Y., Y. Wu, Z.L., and Y.R.; writing – review & editing, all authors; supervision, Z.L., Y.R., and Y.Z.; and funding acquisition, Z.L.

## Declaration of interests

The authors declare no competing interests.

## STAR★Methods

### Key resources table

**Table:** 

REAGENT or RESOURCE	SOURCE	IDENTIFIER
**Antibodies**		

anti-β-actin	Cell Signaling Technology	Cat# 4970; RRID: AB_2223172
anti-NRF2	Abcam	Cat# ab62352; RRID: AB_944418
anti-NRF1	Santa Cruz Biotechnology	Cat# sc-28379; RRID: AB_628042
anti-NRF3	ABclonal	Cat# A15761; RRID: AB_2763179
anti-ubiquitin	Cell Signaling Technology	Cat# 43124; RRID: AB_2799235
anti-CUL3	Cell Signaling Technology	Cat# 10450; RRID: AB_2943632
anti-KEAP1	Abclonal	Cat# A25951; RRID: AB_3711254
Normal Rabbit IgG	Cell Signaling Technology	Cat# 2729; RRID: AB_1031062
anti-RNA Pol II pSer2 CTD	Abcam	Cat# ab238146; RRID: AB_3752720

**Biological samples**		

FFPE ESCC tissues from two previously established patient cohorts	Cui et al.^7^; Liu et al.^8^	N/A
Fresh ESCC and LUSC surgical tissues for organoid culture	This paper	N/A

**Chemicals, peptides, and recombinant proteins**		

L013	This paper	N/A
A51	MCE	Cat# HY123954
MLN4924	MCE	Cat# HY-70062
Polybrene	Sigma-Aldrich	Cat# H9268
Puromycin	Sangon Biotech	Cat# A610593
G418	MedChemExpress	Cat# HY-17561
Recombinant NRF2 proteins	This paper	N/A

**Critical commercial assays**		

Cell Counting Kit-8 (CCK-8)	Dojindo	CK04
FastKing RT Kit	TIANGEN	KR128
PowerUp SYBR Green Master Mix	Applied Biosystems	A25742
SimpleChIP Plus Enzymatic Chromatin IP Kit	Cell Signaling Technology	9005
BCA Protein Assay	Thermo Fisher Scientific	A55864

**Deposited data**		

RNA-seq of 5 SCC PDOs	This paper	GEO: GSE324866

**Experimental models: Cell lines**		

Human ESCC cell lines	Gift from Dr. Yutaka Shimada (Kyoto University)	N/A
Het-1A	ATCC	CRL-2692; RRID: CVCL_3702
ZEC134	Gift from Dr. Dan Su (Zhejiang Cancer Hospital)	N/A

**Experimental models: Organisms/strains**		

ESCC and LUSC organoids	This paper	Primary organoids established from surgical samples
BALB/c nude mice	HFK Bioscience	N/A

**Oligonucleotides**		

See Table S3 for shRNA sequences targeting NFE2L2	This paper	N/A
See Table S4 for qPCR primers	This paper	N/A
See Table S5 for ChIP-qPCR primers for NFE2L2 promoter and gene body	This paper	N/A

**Software and algorithms**		

GraphPad Prism 7.0	GraphPad Software	https://www.graphpad.com/
ImageJ	NIH	https://imagej.nih.gov/ij/

### Experimental model and study participant details

#### Patient samples

This study was approved by the Research Ethics Committee of the Chinese Academy of Medical Sciences Cancer Hospital (Protocol No. NCC2024C-247). Written informed consent was obtained from all enrolled patients. The two ESCC patient cohorts were previously established in our prior work.^7^^,^^8^ For the current analysis, NRF2 IHC staining was conducted on FFPE tissues from a subset of patients in the cohorts, and their prognostic survival follow-up time was updated. Specifically, 85 and 286 patients with valid NRF2 IHC results and complete survival data were included in the two cohorts, respectively. Detailed clinical and pathological characteristics of the two cohorts are summarized in Tables S1 and S2, respectively.

#### Cell lines and cell culture

Human ESCC cell lines were kindly provided by Dr. Yutaka Shimada (Kyoto University, Kyoto, Japan). Het-1A cells were obtained from the American Type Culture Collection (ATCC, USA), and ZEC134 cells were generously provided by Dr. Dan Su (Zhejiang Cancer Hospital, China). All cell lines were authenticated by short tandem repeat (STR) profiling upon receipt in our laboratory to confirm cell identity. Cells were routinely monitored for mycoplasma contamination using PCR analysis and were mycoplasma-free throughout the study. ESCC cells were cultured in RPMI 1640 medium (HyClone, USA) supplemented with 10% fetal bovine serum (FBS; Gibco, USA). Het-1A cells were maintained in DMEM (Corning, USA) supplemented with 10% FBS. Cells were cultured under standard conditions at 37°C in 5% CO2.

#### Organoid models

Fresh tumor tissues from patients with ESCC or LUSC undergoing surgical resection were collected after written informed consent under a protocol approved by the Research Ethics Committee of the Chinese Academy of Medical Sciences Cancer Hospital (Protocol No. NCC2024C-247). Fresh tissues were mechanically and enzymatically dissociated and used to establish patient-derived organoids in commercial organoid medium (bioGenous, China) according to the manufacturer’s instructions. Organoids were cultured at 37°C in a humidified incubator with 5% CO_2_, with medium refreshed every 3 days. Organoids were passaged after 7–10 days of culture. Histologic evaluation by H&E staining and NRF2 IHC was used to confirm organoid identity and NRF2 expression before drug testing.

#### Animal models

For cell line-derived xenograft experiments, 6-week-old male BALB/c nude mice were used for subcutaneous implantation of KYSE150 cells. All mice were housed under specific pathogen-free (SPF) conditions with a 12 h light/dark cycle, constant temperature (22 ± 2°C) and humidity (50%–60%). Mice had free access to standard laboratory chow and sterile drinking water. All animal procedures were approved by the Animal Care and Use Committee of the Chinese Academy of Medical Sciences Cancer Hospital (Protocol No. NCC2024A154).

### Method details

#### Chemical reagents, equipment and synthetic route for L013

All commercial materials (Alfa Aesar, Aladdin, J&K Chemical LTD, Energy Chemical, etc.) were used without further purification. All solvents were analytical grade. The A51 was prepared by the procedure reported by Yinon Ben-Neriah in 2018.^31^ The CRBN ligands were prepared by the procedure reported by Christian Steinebach in 2022.^46^ The ^1^H NMR and ^13^C NMR spectra were recorded on a Bruker AVANCE^Ⅲ^400 MHz spectrometer in CDCl_3_, DMSO-*d*_*6*_ using solvent peak as a standard. All ^13^C NMR spectra were recorded with complete proton decoupling. Low-resolution mass spectral analyses were performed with an Agilent 6340 or Waters AQUITY UPLC™/MS. All reactions were carried out in sealed tube with Teflon cap. Analytical TLC was performed on Yantai Chemical Industry Research Institute silica gel 60 F254 plates and flash column chromatography was performed on Qingdao Haiyang Chemical Co. Ltd silica gel 60 (200-300mesh).

**Figure:**
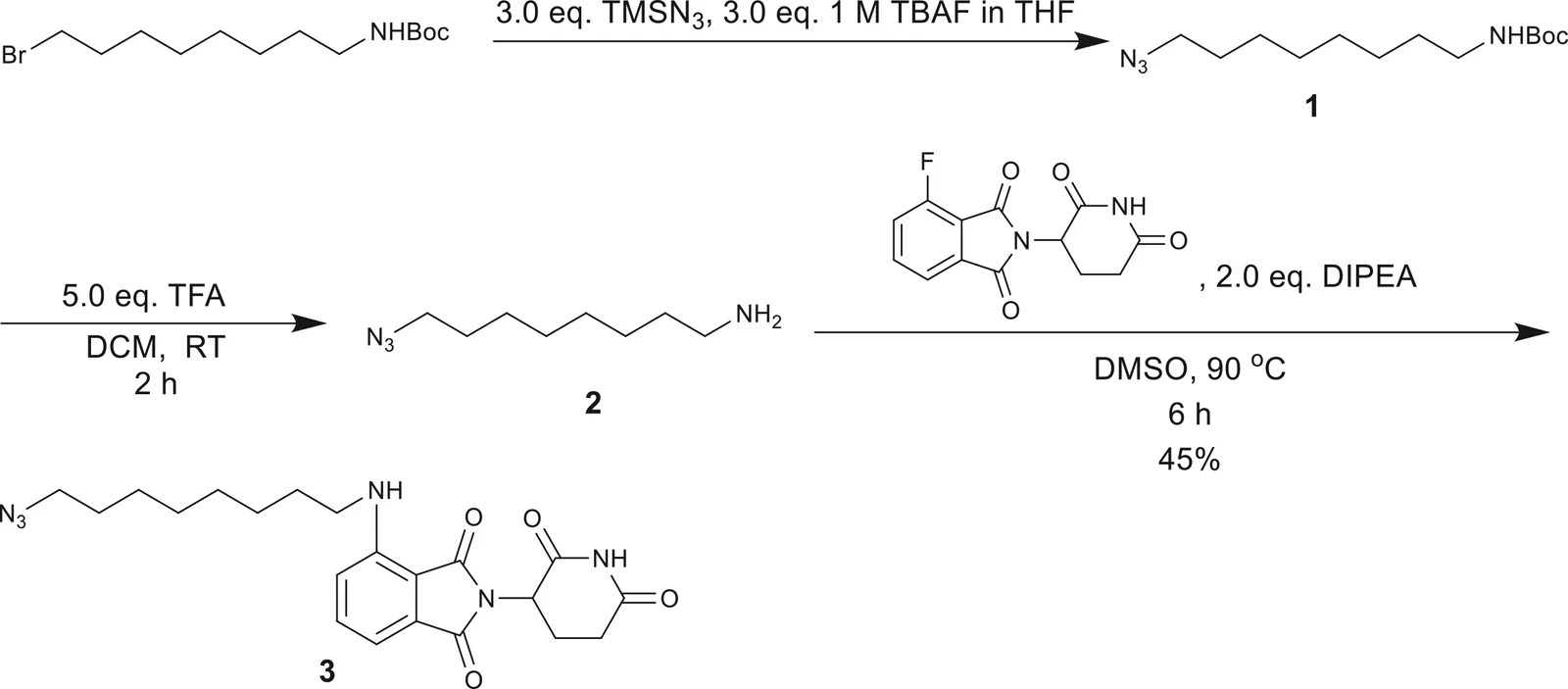

#### Synthesis of compound 3

*Tert*-Butyl (8-bromooctyl)carbamate (3.05 g, 9.90 mmol, 1.00 equiv) was dissolved in tetrahydrofuran (THF, 25 mL). Trimethylsilyl azide (3.90 mL, 29.7 mmol, 3.00 equiv) and a 1.0 M solution of tetrabutylammonium fluoride (TBAF) in THF (29.7 mL, 29.7 mmol, 3.00 equiv) were added sequentially at room temperature. The reaction mixture was heated to 60°C and stirred for 6 h. Upon completion, the mixture was cooled to room temperature, poured into saturated aqueous ammonium chloride solution (50 mL), and extracted with ethyl acetate (50 mL × 3). The combined organic phases were washed sequentially with saturated brine (30 mL), dried over anhydrous sodium sulfate, filtered, and concentrated under reduced pressure. The residue was purified by silica gel column chromatography (eluent: petroleum ether:ethyl acetate = 1:5) to afford *tert*-butyl (8-azidooctyl)carbamate as a colorless oil (Compound **1**, 2.17 g, 8.02 mmol, 81% yield). ^1^H NMR (400 MHz, CDCl_3_) δ 4.54 (s, 1H), 3.26 (t, *J* = 7.0 Hz, 2H), 3.11 (q, *J* = 6.9 Hz, 2H), 1.59 (q, *J* = 7.0 Hz, 2H), 1.45 (s, 9H), 1.34–1.26 (m, 10H).

The above compound **1** (2.17 g, 8.02 mmol) was dissolved in dichloromethane (20 mL), and trifluoroacetic acid (TFA, 3.1 mL, 40.1 mmol, 5.00 equiv) was added. The mixture was stirred at room temperature for 2 h. The mixture was neutralized with N,N-diisopropylethylamine (DIPEA) to pH ≈ 7.0 (monitored by pH paper), and the solvent and excess TFA were removed by rotary evaporation under reduced pressure. The resulting free amine (compound **2**) was dissolved in anhydrous DMSO (80 mL). DIPEA (2.8 mL, 16.0 mmol, 2.00 equiv) and 2-(2,6-dioxopiperidin-3-yl)-4-fluoro-1H-isoindole-1,3(2H)-dione (2.66 g, 9.63 mmol, 1.20 equiv) were added sequentially. The reaction system was heated to 90°C and stirred for 6 h. After cooling, the mixture was diluted with saturated brine (30 mL) and extracted with ethyl acetate (EA, 50 mL × 3). The combined organic phases were washed with saturated brine (30 mL × 3), dried over anhydrous sodium sulfate, filtered, and concentrated under reduced pressure. The residue was purified by silica gel column chromatography (eluent: dichloromethane:methanol = 80:1) to afford the target compound **3** as a yellow solid (1.50 g, 3.61 mmol, 45% yield). ^1^H NMR (400 MHz, CDCl_3_) δ 8.13 (s, 1H), 7.51 (t, *J* = 7.9 Hz, 1H), 7.11 (d, *J* = 7.1 Hz, 1H), 6.90 (d, *J* = 8.5 Hz, 1H), 6.25 (s, 1H), 4.94 (dd, *J* = 12.1, 5.4 Hz, 1H), 3.29 (q, *J* = 5.1 Hz, 4H), 2.96–2.80 (m, 2H), 2.84–2.70 (m, 2H), 1.69 (t, *J* = 7.2 Hz, 2H), 1.40 (m, 10H).

**Figure:**
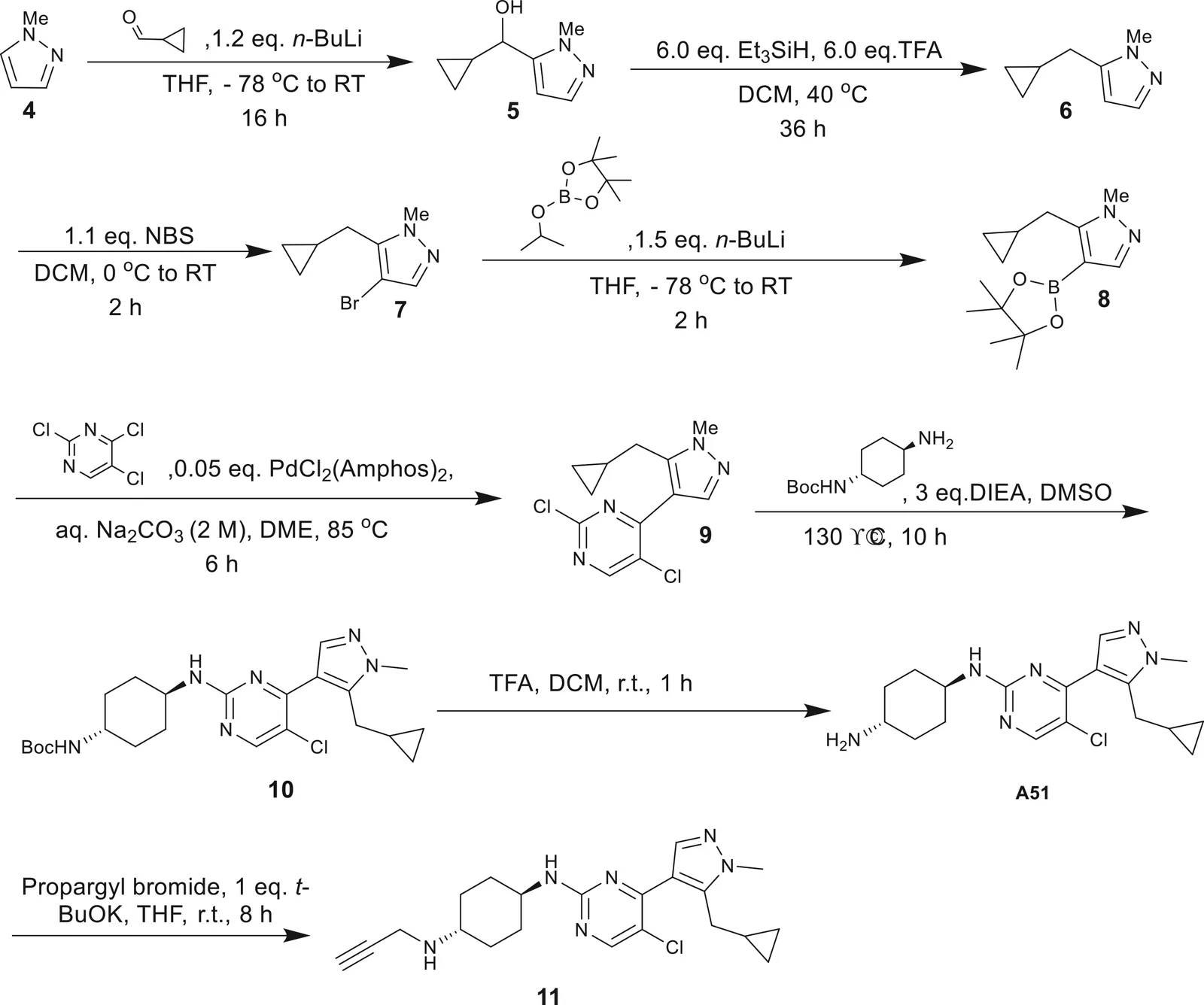

#### Synthesis of compound 11

*N*-Methylpyrazole (3.33 g, 40.6 mmol, 1.00 equiv) was dissolved in super-dry tetrahydrofuran (THF, 67 mL). After argon protection and drying, *n*-butyllithium (*n*-BuLi, 2.5 M, 19.5 mL, 1.20 equiv) was added dropwise at −78°C. After the addition, the mixture was stirred at this temperature for 1 h. Subsequently, a solution of cyclopropanecarbaldehyde (3.42 g, 48.7 mmol, 1.20 equiv) in super-dry THF (33 mL) was added dropwise to the reaction system at −78°C. Upon completion of addition, the reaction solution was slowly warmed to room temperature (approx. 20°C) and stirred for 16 h. After the reaction completed, the reaction mixture was poured into saturated aqueous ammonium chloride solution (125 mL) and stirred for 10 min. The aqueous phase was extracted with ethyl acetate (50 mL × 3). The combined organic phases were washed with saturated brine (50 mL), dried over anhydrous sodium sulfate, filtered, and concentrated under reduced pressure. The resulting residue was purified by silica gel column chromatography (eluent: gradient from petroleum ether:ethyl acetate = 8:1 to pure ethyl acetate) to afford target compound **5** (5.00 g, 32.85 mmol, 80% yield) as a colorless oily liquid. ^1^H NMR (400 MHz, CDCl_3_) δ 6.97 (s, 1H), 5.98 (s, 1H), 5.37 (s, 1H), 3.82 (d, *J* = 7.8 Hz, 1H), 3.53 (s, 3H), 1.03 (s, 1H), 0.36–0.00 (m, 4H).

Compound **5** (2.79 g, 18.35 mmol, 1.00 equiv), trifluoroacetic acid (TFA, 12.55 g, 110.1 mmol, 8.42 mL, 6.00 equiv), and triethylsilane (12.80 g, 110.1 mmol, 17.5 mL, 6.00 equiv) were dissolved in dichloromethane (DCM, 280 mL) and stirred at 40°C for 36 h. Upon completion, the mixture was adjusted to pH ≈ 8 with saturated aqueous sodium bicarbonate solution, and the layers were separated. The organic phase was concentrated under reduced pressure, and the residue was purified by silica gel column chromatography (eluent: petroleum ether:ethyl acetate = 20:1 to 8:1) to afford compound **6** (1.00 g, 7.34 mmol, 40% yield) as a yellow oil. ^1^H NMR (400 MHz, CDCl_3_) δ 7.35 (d, *J* = 4.0 Hz, 1H), 6.17 (s, 1H), 3.80 (s, 3H), 2.54 (d, *J* = 6.7 Hz, 2H), 1.07–0.99 (m, 1H), 0.64–0.55 (m, 2H), 0.22 (t, *J* = 5.1 Hz, 2H).

Compound **6** (0.559 g, 4.11 mmol, 1.00 equiv) was dissolved in dichloromethane (DCM, 5.6 mL). At 0°C, *N*-bromosuccinimide (0.805 g, 4.52 mmol, 1.10 equiv) was added. After addition, the reaction mixture was warmed to room temperature and stirred for 2 h. The reaction solution was concentrated under reduced pressure and purified by silica gel column chromatography (eluent: petroleum ether:ethyl acetate = 20:1 to 10:1) to afford compound **7** (0.8 g, 3.72 mmol, 90% yield) as a yellow oil.^1^H NMR (400 MHz, CDCl_3_) δ 7.41 (s, 1H), 3.88 (d, *J* = 1.1 Hz, 3H), 2.66 (d, *J* = 6.6 Hz, 2H), 1.02–0.92 (m, 1H), 0.53 (p, *J* = 6.3 Hz, 2H), 0.29 (t, *J* = 5.1 Hz, 2H).

Compound **7** (0.743 g, 3.45 mmol, 1.00 equiv) was dissolved in super-dry tetrahydrofuran (THF, 15 mL). After argon protection and drying treatment, *n*-butyllithium (*n*-BuLi, 2.0 M, 2.59 mL, 1.50 equiv) was added dropwise at −78°C. After addition, stirring was continued at this temperature for 1 h. Subsequently, a solution of 2-isopropoxy-4,4,5,5-tetramethyl-1,3,2-dioxaborolane (1.29 g, 6.91 mmol, 2.00 equiv) in super-dry THF (1.5 mL) was added dropwise to the reaction system. After addition, the reaction was slowly warmed to room temperature (approx. 20°C) and stirred for 1 h. After completion, the reaction was quenched with saturated aqueous ammonium chloride solution (15 mL) and extracted with ethyl acetate (25 mL × 3). The organic phase was concentrated under reduced pressure and purified by silica gel column chromatography (eluent: petroleum ether:ethyl acetate = 20:1 to 10:1) to afford compound **8** (0.552 g, 1.73 mmol, 50% yield) as a colorless oil. ^1^H NMR (400 MHz, CDCl_3_) δ 7.68 (s, 1H), 3.88 (s, 3H), 2.82 (d, *J* = 6.7 Hz, 2H), 1.30 (s, 12H), 1.01–0.91 (m, 1H), 0.47–0.40 (m, 2H), 0.32–0.25 (m, 2H).

Compound **8** (335 mg, 1.28 mmol, 1.00 equiv) was dissolved in 1,2-dimethoxyethane (6.7 mL). Under argon protection, 2,4,5-trichloropyrimidine (281 mg, 1.54 mmol, 1.20 equiv), 2 M aqueous sodium carbonate solution (1.41 mL, 2.82 mmol, 2.20 equiv), and catalyst PdCl_2_(Amphos)_2_ (45.4 mg, 0.064 mmol, 0.05 equiv) were added sequentially. The reaction mixture was heated to 85°C under a nitrogen atmosphere and stirred for 6 h. Then the reaction mixture was cooled to room temperature, the mixture was diluted with saturated brine (34 mL) and extracted with ethyl acetate (34 mL × 3). The organic phase was concentrated under reduced pressure and purified by silica gel column chromatography (eluent: petroleum ether:ethyl acetate = 5:1) to afford compound **9** (235 mg, 0.706 mmol, 55% yield) as a yellow oil. ^1^H NMR (400 MHz, Chloroform-*d*) δ 8.53 (s, 1H), 8.30 (s, 1H), 3.96 (s, 3H), 3.11 (d, *J* = 6.7 Hz, 2H), 1.11–1.05 (m, 1H), 0.54–0.49 (m, 2H), 0.37–0.33 (m, 2H).

To a reaction flask, compound **9** (25 mg, 0.09 mmol), *trans*-4-(Boc-amino)cyclohexylamine (22 mg, 0.11 mmol), and *N,N*-diisopropylethylamine (0.046 mL, 0.27 mmol) were added sequentially, followed by dimethyl sulfoxide (0.5 mL) to fully dissolve the reactants. The reaction system was heated to 130°C and maintained at this temperature for 10 h. Upon completion, the reaction mixture was extracted with ethyl acetate. The organic layer was dried over anhydrous sodium sulfate and concentrated under vacuum to remove the solvent. Purification was performed using column chromatography (petroleum ether:ethyl acetate = 1:1). Compound **10** was obtained as white solid with a yield of 44.2%. ^1^H NMR (400 MHz, Chloroform-*d*) δ 8.22 (s, 1H), 8.13 (s, 1H), 4.91–4.89 (d, *J* = 8.3 Hz, 1H), 3.93 (s, 3H), 3.82–3.75 (m, 1H), 3.07–3.05 (d, *J* = 8.0 Hz, 2H), 2.18–2.12 (m, 2H), 2.12–2.07 (m, 2H), 1.47 (s, 9H), 1.34–1.29 (m, 4H), 1.07–1.00 (m, 1H), 0.53–0.46 (m, 2H), 0.22–0.17 (m, 2H).

Compound **10** and trifluoroacetic acid (TFA, 0.1 mL) were added to a reaction flask, followed by dichloromethane (DCM, 0.5 mL) to fully dissolve the reactants. The reaction system was stirred at room temperature for 1 h. Upon completion, the reaction mixture was concentrated under reduced pressure to afford the crude intermediate, referred to as compound **A51**. Next, to the flask containing the concentrate, 3-bromopropyne (4.1 mg, 0.035 mmol) and potassium *tert*-butoxide (20 mg, 0.16 mmol) were added, followed by tetrahydrofuran (THF, 0.5 mL) to fully dissolve the reactants. The reaction system was reacted at room temperature for 8 h. Upon completion, the reaction mixture was extracted with ethyl acetate. The organic layer was dried over anhydrous sodium sulfate and concentrated under vacuum to remove the solvent. Purification was performed using column chromatography (dichloromethane:methanol = 40:1). Compound **11** was obtained as white solid with a yield of 32.3%. ^1^H NMR (400 MHz, Chloroform-*d*) δ 8.21 (s, 1H), 8.15 (s, 1H), 5.04 (s, 1H), 3.93 (s, 3H), 3.51 (d, *J* = 2.5 Hz, 2H), 3.09–3.07 (d, *J* = 8.0 Hz, 2H), 2.26–2.24 (t, *J* = 4.0 Hz, 1H), 2.18 (s, 1H), 2.00 (s, 2H), 1.31–1.27 (m, 8H), 1.07–1.01 (m, 1H), 0.53–0.46 (m, 2H), 0.21–0.16 (m, 2H).

**Figure:**
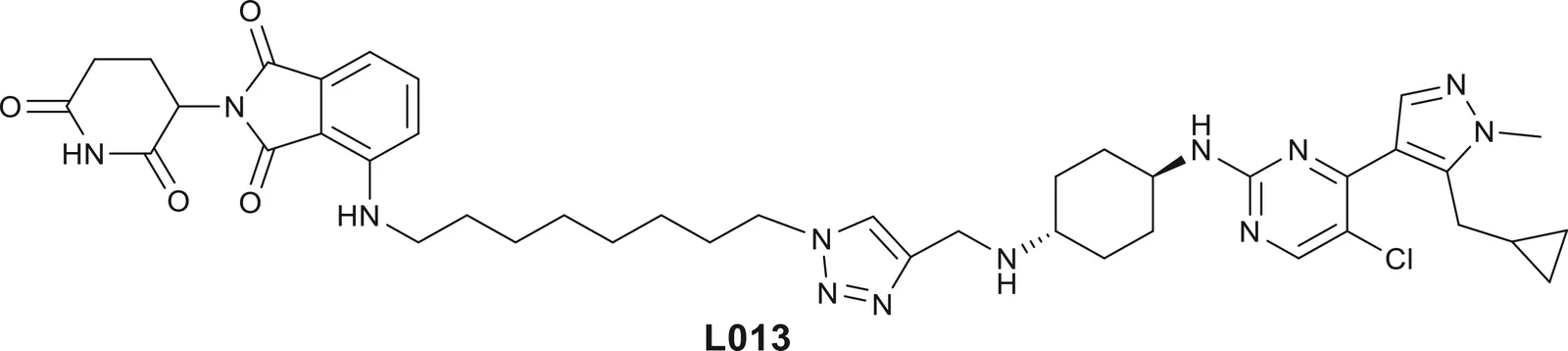

**4-((8-(4-((((1*r*,4*r*)-4-((5-chloro-4-(5-(cyclopropylmethyl)-1-methyl-1*H*-pyrazol-4-yl)pyrimidin-2-yl)amino)cyclohexyl)amino)methyl)-1*H*-1,2,3-triazol-1-yl)octyl)amino)-2-(2,6-dioxopiperidin-3-yl)isoindoline-1,3-dione.**

To a stirred solution of compound **3** (0.05 mmol, 20 mg) and compound **11** (0.05 mmol, 21 mg) in *t*-BuOH was added CuSO_4_ (0.025 mmol, 4 mg), sodium ascorbate (0.15 mmol, 30 mg) and water (10% to *t*-BuOH). The reaction mixture was stirred at 70°C under argon for 4 h. After completion, the mixture was diluted with ethyl acetate and washed with brine, dried over Na_2_SO_4_, filtered and concentrated. The crude residue was purified by column chromatography (DCM:MeOH = 30:1) to afford compound **L013** (12 mg, 30% yield). ^1^H-NMR(400MHz, DMSO-*d*_*6*_, ppm): 11.01 (s, 1H), 8.28 (s, 1H), 8.25 (s, 1H), 8.02 (s, 1H), 7.59 (t, J = 7.60 Hz, 1H), 7.27 (s, 1H), 7.09 (d, J = 8.68 Hz, 1H), 7.02 (d, J = 7.00 Hz, 1H), 6.51 (s, 1H), 5.07(dd, J = 4.84 Hz, J = 12.72 Hz, 1H), 4.41 (t, J = 6.60 Hz, 2H), 4.21 (s, 2H), 3.86 (s, 3H), 3.68 (s, 1H), 3.29 (d, J = 6.24 Hz, 2H), 3.06 (d, J = 5.88 Hz, 2H), 3.33–2.50 (m, 3H), 2.17 (d, J = 11.12 Hz, 2H), 2.04 (m, 3H), 1.82 (t, J = 6.84 Hz, 2H), 1.56–1.23 (m, 16H), 0.99 (s, 1H), 0.42 (s, 2H), 0.16 (s, 2H). LC-MS: calculated for C_42_H_54_ClN_12_O_4_ [M + H]^+^: 825.40, found 825.27.

#### Plasmids and antibodies

The following primary antibodies were used for immunoblotting analysis: anti-β-actin (1:2000, Cat# 4970, Cell Signaling Technology, USA), anti-NRF2 (1:1000, Cat# ab62352, Abcam, UK), anti-NRF1 (1:500, Cat# sc-28379, Santa Cruz Biotechnology, USA), anti-NRF3 (1:1000, Cat# A15761, ABclonal, China), anti-CUL3 (1:1000, Cat# 10450, Cell Signaling Technology, USA), anti-KEAP1(1:1000, Cat# A25951, ABclonal, China), anti-ubiquitin (1:1000, Cat# 43124, Cell Signaling Technology), anti-RNA Pol II pSer2 CTD antibody (ab238146, Abcam, UK), and Normal Rabbit IgG (1:1000, Cat# 2729, Cell Signaling Technology).

Full-length wild-type or mutant *NFE2L2* cDNA was subcloned into the pLVX-IRES-neo lentiviral vector for ectopic expression. The full-length *CRBN* cDNA was subcloned into the Flag-tagged pLVX-IRES-neo lentiviral vector for ectopic expression. Full-length *NFE2L2* cDNA was subcloned into the pET28a vector for *in vitro* protein purification. shRNA sequences targeting *NFE2L2* were cloned into the pSIH1-puro vector to establish stable knockdown cell lines. The shRNA target sequences are provided in Table S3.

#### Cell proliferation assay

Cells were seeded in 96-well plates at a density of 3 × 10^3^ cells per well and cultured for 12 h to allow adhesion. For the CCK-8 assay, 10 μL of CCK-8 reagent (Dojindo, Japan) was added to each well. After 1 h of incubation at 37°C, absorbance was measured at 450 nm using a microplate reader (BioTek, USA). For drug treatment experiments, cells were treated with indicated drugs or vehicles and monitored dynamically using the Incucyte S3 Live-Cell Analysis System (Sartorius, Germany). Cell confluence was quantified by capturing images every 2–6 h under standard culture conditions (37°C, 5% CO_2_). Data were analyzed using Incucyte S3 software to generate time-dependent proliferation curves. Experiments were performed with *n* = 6 independent biological replicates.

#### Colony formation assay

Cells were plated in 6-well plates at 1.5 × 10^3^ cells per well and cultured in complete medium for 10–14 days. Colonies were fixed with 4% paraformaldehyde for 15 min and stained with 0.1% crystal violet for 30 min. Colonies were manually counted under a light microscope. Experiments were performed with *n* = 3 independent biological replicates.

#### Immunoblotting

Total protein was extracted using RIPA lysis buffer (CWBIO, China) supplemented with protease inhibitors (Roche, Switzerland). Protein concentrations were quantified via BCA assay (Thermo Fisher, USA). Equal amounts of protein (20 μg per lane) were separated by 10% SDS-PAGE and transferred to PVDF membranes (Millipore, USA). Membranes were blocked with 5% non-fat milk for 1 h and incubated overnight at 4°C with primary antibodies. After washing, membranes were probed with HRP-conjugated secondary antibodies for 1 h. Signals were visualized using ECL substrate (Thermo Fisher, USA) and analyzed with ImageJ software.

For co-immunoprecipitation assays, cells were lysed with mild IP lysis buffer (Beyotime, China) on ice for 30 min. A portion of the supernatant was reserved as the input control. The remaining supernatant was incubated with Anti-Flag magnetic beads or Protein A/G magnetic beads overnight at 4°C with gentle rotation. After incubation, the beads were washed thoroughly with pre-cooled TBST or PBST buffer three times to remove non-specific binding proteins. The immunoprecipitated proteins were eluted by boiling the beads in SDS loading buffer for 10 min. Finally, the eluted protein samples were subjected to Western blot analysis as described above to detect target protein interactions.

#### RNA extraction and quantitative RT-PCR

Total RNA was isolated using TRIzol reagent (Invitrogen, USA) and reverse-transcribed into cDNA with FastKing RT Kit (TIANGEN, China). qPCR was performed using PowerUp SYBR Green Master Mix (Applied Biosystems, USA) on a QuantStudio5 Real-Time PCR System (Applied Biosystems, USA). The 2^−ΔΔCt^ method was applied to quantify relative mRNA levels, normalized to the housekeeping gene GAPDH. Primer sequences are listed in Table S4.

#### High-content screening system for degrader screening

To identify degrader molecules targeting NRF2, we first constructed an overexpression plasmid encoding a GFP-NRF2 fusion protein. The plasmid was packaged into lentivirus and transduced into KYSE150 cell line. Stable GFP-NRF2-expressing cell lines were established through antibiotic selection and further enriched via flow cytometry sorting of GFP-high cells. For screening, GFP fluorescence quenching (indicative of NRF2 degradation) was monitored using a high-content imaging system (Molecular Devices, USA). Notably, degradation of the GFP-NRF2 fusion protein resulted in a transition from diffuse fluorescence to punctate fluorescent granules. To quantify degradation efficiency, we developed a custom analysis protocol that measured the number of fluorescent granules per GFP-positive cell as the primary readout, enabling comparative evaluation of degrader candidates.

#### Drug affinity responsive target stability assay

Purified recombinant NRF2 and BSA proteins were incubated with dialysis buffer and aliquoted into 1.5 mL tubes. Candidate drugs or DMSO (vehicle control) were added to the mixtures, followed by treatment with Pronase E at 40°C for 20 min. Protein degradation was assessed by SDS-PAGE and immunoblotting. Degrader molecules exhibiting binding affinity for NRF2 were identified by their ability to protect NRF2 (but not BSA) from Pronase-mediated degradation.

#### Recombinant protein expression

Full-length human *NFE2L2* cloned into pET28a was transformed into chemically competent E. coli BL21(DE3) cells by heat shock, and transformants were selected on LB agar plates containing 50 μg/mL kanamycin. For small-scale expression testing, single colonies were inoculated into kanamycin-containing LB medium and cultured at 37°C until OD600 reached 0.6–0.8. Protein expression was induced with 0.6 mM IPTG at 37°C for 4 h. Bacterial pellets were resuspended in lysis buffer (50 mM Tris-HCl, 150 mM NaCl, 5% glycerol, pH 8.0), disrupted by sonication, and separated into total, soluble, and insoluble fractions by centrifugation. Recombinant NRF2 expression was analyzed by SDS-PAGE and Western blot.

#### Surface plasmon resonance

Direct small molecule-NRF2 interactions were analyzed using a Biacore 8 K system (Cytiva, USA) at 25°C. Running buffer was PBS-P (phosphate-buffered saline containing 0.005% (v/v) Tween 20) supplemented with 5% DMSO. Full-length NRF2 protein was immobilized on a Series S CM5 chip (Cytiva) via amine coupling to a surface density >5000 RU; an uncoated reference flow cell was used for background subtraction. The “LMW multi-cycle kinetics/affinity” method was used per the manufacturer’s guidelines. A startup step (30 μL/min flow rate, 120 s contact, 60 s dissociation over both flow cells) was followed by 50% DMSO washing. Solvent correction (2 cycles) was applied at the start, every 48 cycles, and end to mitigate DMSO interference. Small molecules (various concentrations) were injected as variable solutions (30 μL/min, 90 s contact, 90 s dissociation), with 50% DMSO washing post-injection. Chip regeneration was performed with 10 mM glycine-HCl (pH 2.0) at 30 μL/min for 30 s. Kinetic sensorgrams (Biacore Insight Evaluation software, Cytiva) were double-referenced (reference flow cell + blank buffer subtraction). Equilibrium dissociation constants (K_D_) were calculated via global fitting of corrected sensorgrams to a 1:1 Langmuir binding model.

#### Isothermal titration calorimetry

The ITC experiment was performed on a NANO ITC instrument (TA instruments, China). After cleaning the sample cell and titration needle, the full-length NRF2 protein was carefully injected into the sample cell using a micro-syringe without any bubbles at the concentration of 3 μM for L013 titration. L013 (30 μM) was filled into a 60 μL titration syringe. All titrations were carried out at 25°C with 20 sequential injections of the ligand into the sample cell, and an interval of 200 s was set between each injection. Deionized water was added to the reference cell as a heat balance control. The obtained titration curves were analyzed, and the binding parameters including the equilibrium dissociation constant (Kd), stoichiometry (n), enthalpy change (ΔH) and entropy change (ΔS) were calculated using the nanoAnalyzer software.

#### Microscale thermophoresis

The MST measurements for the binding of L013 to full-length NRF2 were performed on a Monolith X instrument (NanoTemper, Germany) following the manufacturer’s standard protocols. Briefly, the full-length NRF2 protein was first buffer-exchanged into the Labeling Buffer from the RED-NHS labeling kit (NanoTemper, MO-L011) and adjusted to a final concentration of 5 μM. The protein was then fluorescently labeled with the RED-NHS dye (100 μL, 3× the protein concentration) by incubating the mixture for 30 min at room temperature in the dark. Excess unreacted dye was removed using the size-exclusion column from the kit, and the labeled NRF2 protein was eluted with Assay Buffer (50 mM HEPES, pH 7.4, 0.05% Tween 20) to obtain the stock solution.

L013 was serially 2-fold diluted into 16 concentration gradients with the aforementioned Assay Buffer. Equal volumes of the labeled NRF2 protein and the gradient-diluted small molecules were mixed and incubated for 20 min at room temperature to reach binding equilibrium. The mixtures were then loaded into standard capillaries (NanoTemper, MO-K022), and the thermophoresis signals were detected on the Monolith X instrument. The equilibrium dissociation constant (Kd) values for the interaction between NRF2 and L013 was calculated using the dedicated NanoTemper analysis software.

#### Chromatin immunoprecipitation-qPCR

Cells were crosslinked with 1% formaldehyde (Sigma, Germany) at room temperature for 10 min. Chromatin preparation and immunoprecipitation were performed using the SimpleChIP Plus Enzymatic Chromatin IP Kit (Cell Signaling Technology, USA) according to the manufacturer’s protocol. Immunoprecipitation was carried out overnight at 4°C with anti-RNA Pol II pSer2 CTD antibody (ab238146, Abcam, UK) or IgG control (#2729, Cell Signaling Technology, USA). Target DNA regions were quantified by qPCR using gene body- and promoter-specific primers for the *NFE2L2* locus (Listed in Table S5). Data were normalized to input DNA and presented as fold enrichment relative to IgG controls.

#### Organoid culture and drug sensitivity assay

When the number and size of organoids reached an ideal state, organoids were collected for H&E staining, as well as immunohistochemical staining to ascertain the tumor organoid formation and the expression levels of the NRF2 protein. To assess the activity of organoids following the addition of L013, organoids were digested and plated into 96-well plates, where different concentrations of L013 were introduced into the culture medium. The activity of organoids post-treatment was measured using the CellTiter-Glo 3D Cell Viability Assay (Promega, USA).

#### Xenograft studies

For cell line-derived xenografts, 1.5×10^6^ KYSE150 cells were subcutaneously injected into 6-week-old male BALB/c nude mice (HFK Bioscience, China), and tumor size was measured every other day using digital calipers with volume calculated as 0.5×length×width.^2^ When tumors reached 100–150 mm^3^, mice were randomized into L013 (10 mg/kg) or vehicle control groups (n = 6–8). Both L013 and the vehicle control were dissolved in 10% NMP +60% PEG400 + 30% PBS, and administered by oral gavage at 10 mg/kg every other day, unless otherwise indicated. Body weight and clinical signs were monitored daily. Euthanasia criteria included tumor volume exceeding 1500 mm^3^ or humane endpoints, such as lethargy or >20% body weight loss. At study termination, tumors were excised, weighed, and fixed for immunohistochemistry (IHC), while blood samples (retro-orbital) and major organs were harvested for analysis. All procedures were approved by the Animal Care and Use Committee of the Chinese Academy of Medical Sciences Cancer Hospital (Protocol No: NCC2024A154).

### Quantification and statistical analysis

Statistical analyses were performed using two-tailed Student’s *t* test for comparisons between two groups and two-way *ANOVA* with multiple comparisons test for comparisons among three or more groups. Survival outcomes were analyzed by Kaplan-Meier curves, and intergroup differences were assessed using the log rank test. Unless otherwise indicated, quantitative data are presented as mean ± SEM or mean ± SD as specified in each figure legend. Statistical calculations were conducted with GraphPad Prism 7.0 (GraphPad Software, USA), and a *p*-value <0.05 was considered statistically significant.

## Supplemental information

Document S1. Figures S1–S6 and Tables S3–S5

Table S1. Clinical information of 85 ESCC patients, related to STAR Methods

Table S2. Clinical information of 286 ESCC patients, related to STAR Methods

Document S2. Article plus supplemental information
