# Supplementary material for: L013 promotes NRF2 degradation in NRFA-subtype esophageal squamous cell carcinoma
Source: Cell Rep Med. 2026 Sep 11;7(10):103046. doi: 10.1016/j.xcrm.2026.103046 (PMC13710151; doi:10.1016/j.xcrm.2026.103046)
Supplement: Supplementary file 1 [file mmc1.pdf]

Cell Reports Medicine, Volume *7*

Supplemental information

L013 promotes NRF2 degradation in NRFA-subtype esophageal squamous cell carcinoma

Xiao Yu, Yue Wu, Xiuyun Sun, Rui Han, Yi Cai, Wei Zeng, Yang Li, Yuhao Wang, Yahui Zhao, Yu Rao, and Zhihua Liu

Supplementary Information

Supplementary Figures

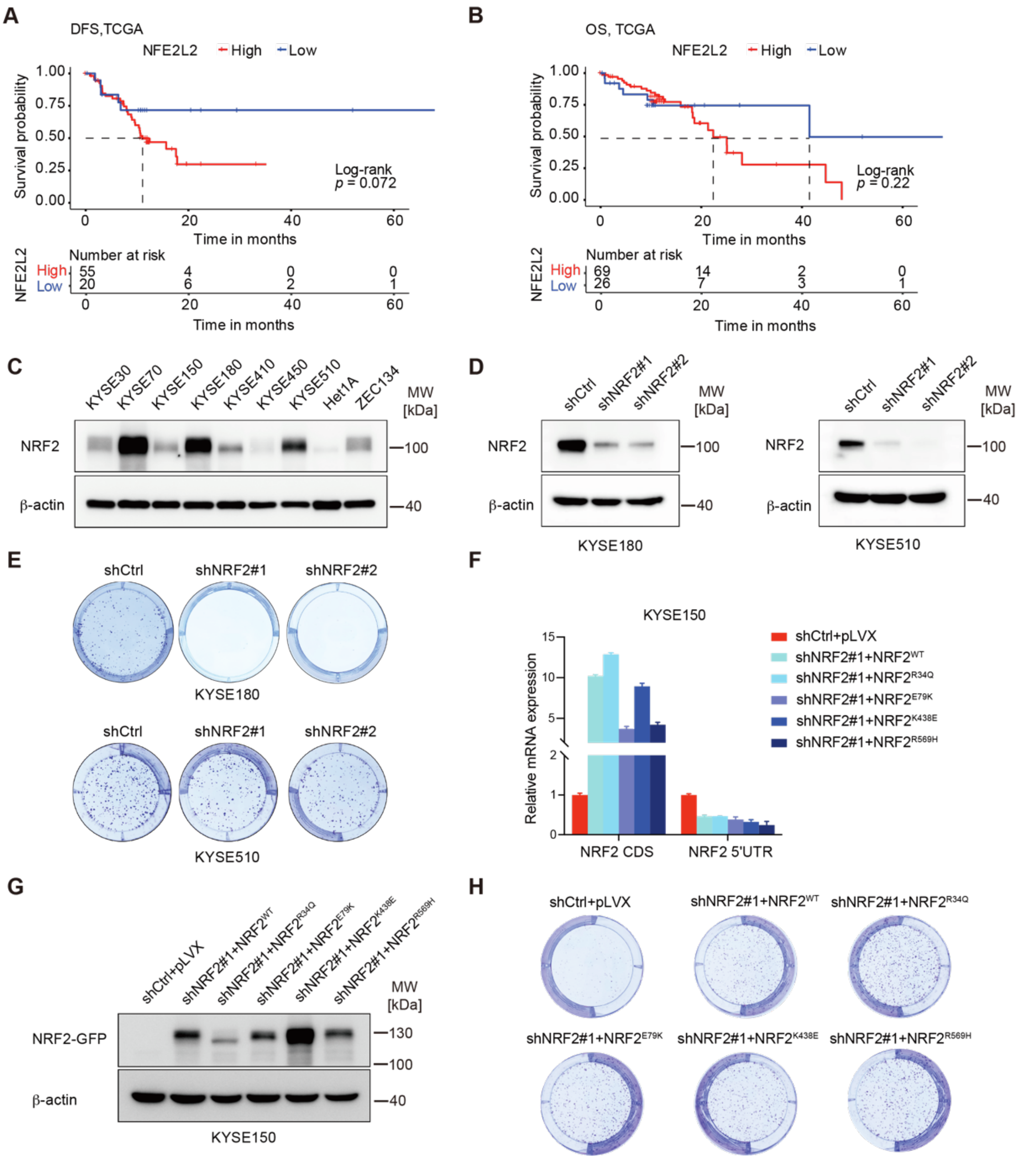

Figure S1. Aberrantly high NRF2 protein expression is associated with poor prognosis and malignant phenotypes in ESCC. Related to Figure 1.

(A, B) Survival analysis using the TCGA ESCC cohort based on the Kaplan-Meier plot databases[S1]. **(C)** Immunoblotting was used to detect NRF2 protein expression levels in various ESCC cell lines. **(D)** Immunoblotting was used to detect NRF2 protein expression in KYSE180 and KYSE510 cells expressing control (shCtrl) or NRF2 shRNAs. **(E)** Representative images of colony formation assay in KYSE180 and KYSE510 cells expressing control (shCtrl) or NRF2 shRNAs. **(F)** qPCR analysis of endogenous NRF2 knockdown and exogenous NRF2 overexpression in KYSE150 cells. **(G)** Immunoblotting was used to detect exogenous NRF2-GFP protein expression in KYSE150 cells expressing shCtrl or NRF2 shRNA#1 followed by overexpression of wild-type and mutant *NFE2L2*. **(H)** Representative images of colony formation assay in KYSE150 cells expressing shCtrl or NRF2 shRNA#1 followed by overexpression of wild-type and mutant *NFE2L2*.

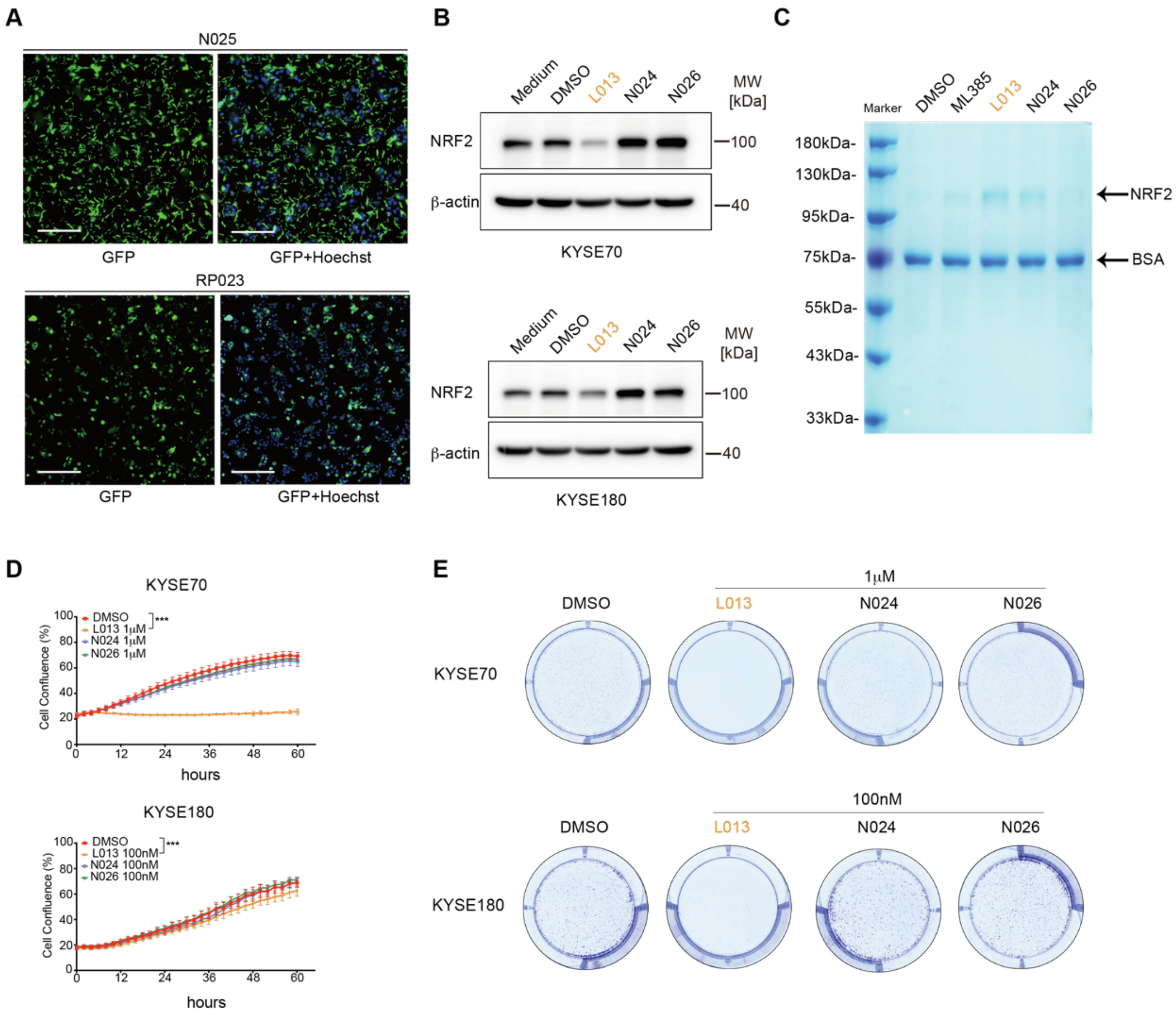

Figure S2. High-content screening identifies L013 as a selective NRF2 degrader. Related to Figure 2.

(A) Representative images captured by high-content imaging system after the addition of indicated degraders with 5 μM for 12 hours. Scale bars represent 100 μm. **(B)** Immunoblotting to detect the levels of endogenous NRF2 protein in KYSE70 and KYSE180 cell lines after treatment with 5 μM degraders for 12 hours. **(C)** The DARTS assay demonstrated that L013 can bind to the NRF2 protein, thereby protecting it from protease degradation. BSA was used as a loading control. **(D)** Cell proliferation curves of KYSE70 and KYSE180 cell lines after treatment with degraders. n = 5 biological replicates. Data are represented as mean ± SD. *** $p < 0.001$ by two-way ANOVA with Tukey's multiple comparisons test. **(E)** Representative images showing the *in vitro* colony formation of KYSE70 and KYSE180 cell lines after treatment with degraders.

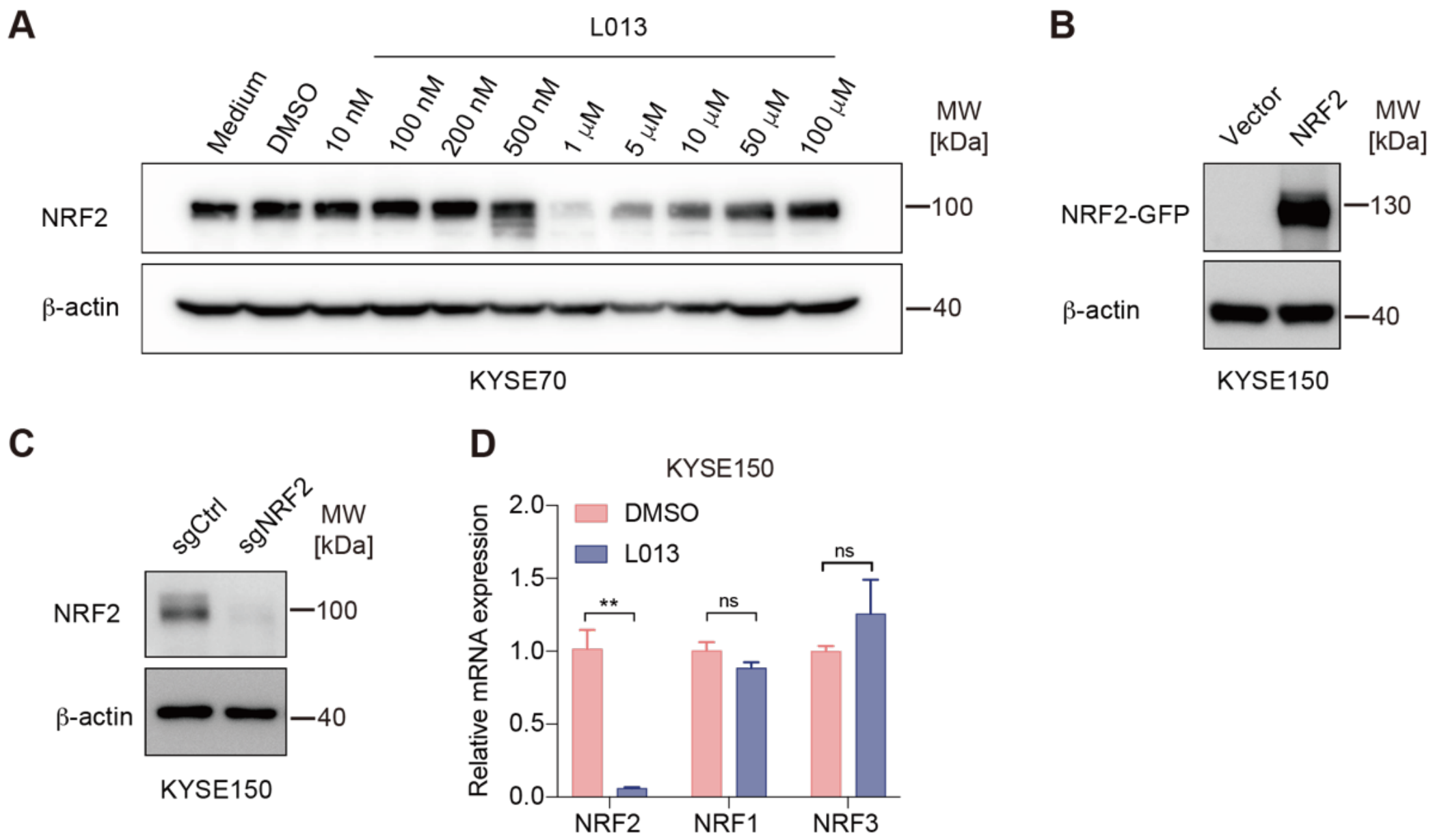

Figure S3. L013 exhibits rapid and selective NRF2 degradation. Related to Figure 3.
(A) Immunoblotting was used to detect the degradation of NRF2 protein by L013 under the indicated concentration for 8 hours in KYSE70 cell lines. **(B)** Immunoblotting detection related to Figure 3D. **(C)** Immunoblotting detection related to Figure 3E. **(D)** qPCR analysis of mRNA expression levels of indicated targets in KYSE150 after addition of 1 μM L013 for 24 hours. n = 3 biological replicates. Data are represented as mean ± SEM. ** $p < 0.01$ by unpaired t-test.

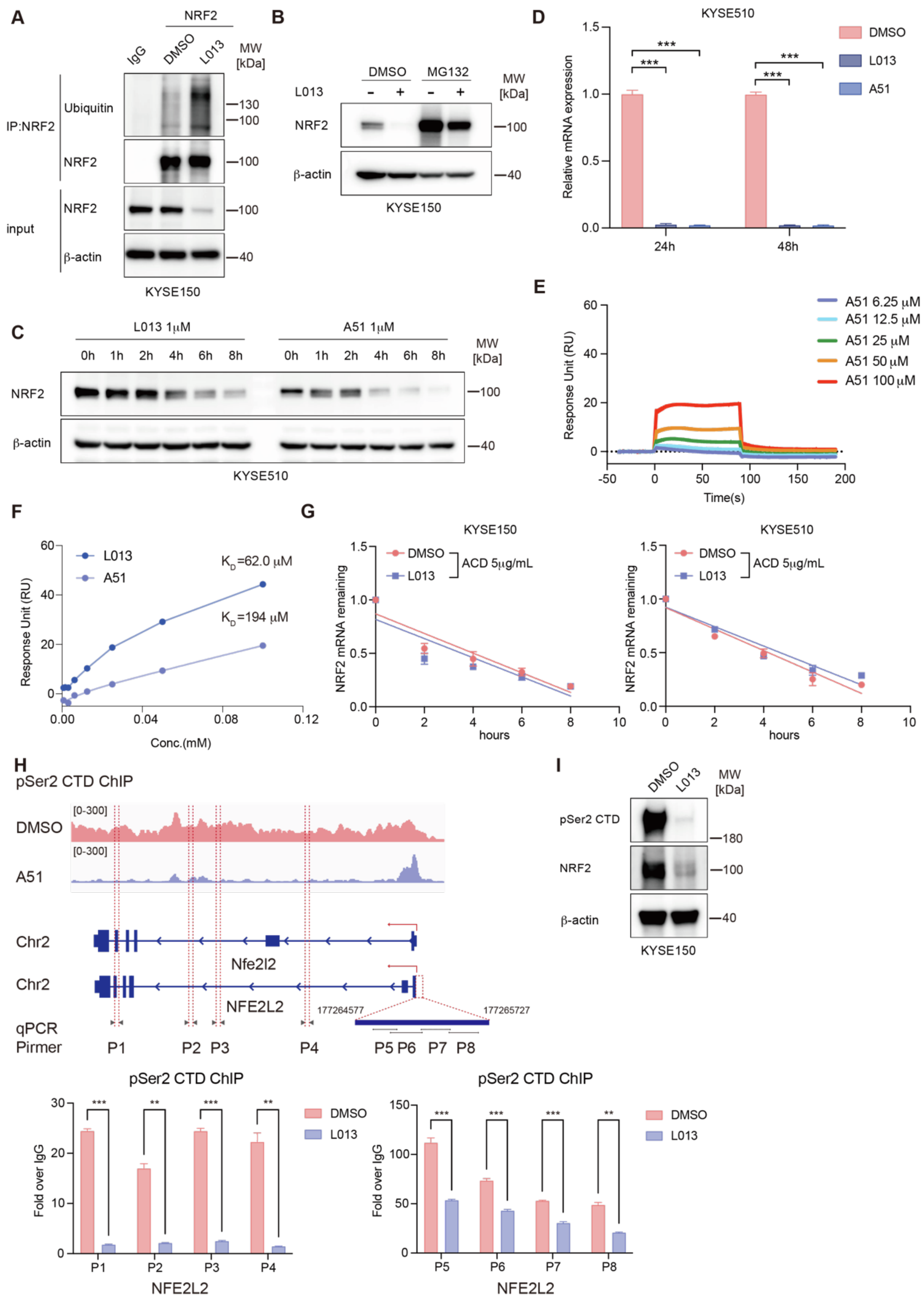

Figure S4. L013 promotes UPS-associated NRF2 reduction while retaining an A51-derived NRF2-suppressive activity. Related to Figure 4.

(A) Immunoblotting was used to detect NRF2 ubiquitination in KYSE150 cells pretreated with 10 μM MG132 for 4 hours followed by treatment with or without 1 μM L013 for an additional 4 hours. IgG was used as negative control. **(B)** Immunoblotting detection for NRF2 after a 2-hour pretreatment with MG132 (2 μM), followed by a 12-hour treatment with L013(1 μM) in KYSE150 cells. **(C)**

Immunoblotting was used to detect NRF2 degradation at the indicated time points after addition of L013 or A51 in KYSE510 cells. **(D)** qPCR analysis of NRF2 expression levels in KYSE510 cells at the indicated time after addition of 1 μM L013 or A51. n = 3 biological replicates, data presented as mean ± SEM. *** $p < 0.001$ by Student's *t* test. **(E, F)** SPR assay revealed the affinity of L013 and A51 for NRF2. The K_D value was analyzed by the Biacore 8k system. **(G)** Time course analysis of *NFE2L2* mRNA stability in KYSE150 and KYSE510 cells treated with DMSO or L013 (1 μM). n = 3 biological replicates, data presented as mean ± SD. **(H)** IGV screenshots showing ChIP-seq profiles for pSer2 CTD around the *Nfe2l2* gene in primary MLL-AF9 AML cells from the public database of GEO: GSE117443. (Top); Schematic representation of the mouse Nfe2l2 gene and the human NFE2L2 gene, showing the position of qPCR primers P1-P8 relative to the gene structure, where primers P1-P4 are located in the gene body region and primers P5-P8 in the TSS region. Red arrows represent the direction of transcription. (Middle); qPCR analysis of pSer2 CTD ChIP in KYSE150 cells treated with DMSO or L013. n = 3 biological replicates, data presented as mean ± SEM. ** $p < 0.01$, *** $p < 0.001$ by unpaired *t* test (Bottom). **(I)** Immunoblotting was used to detect indicated proteins after addition of 1 μM L013 in KYSE150 cells.

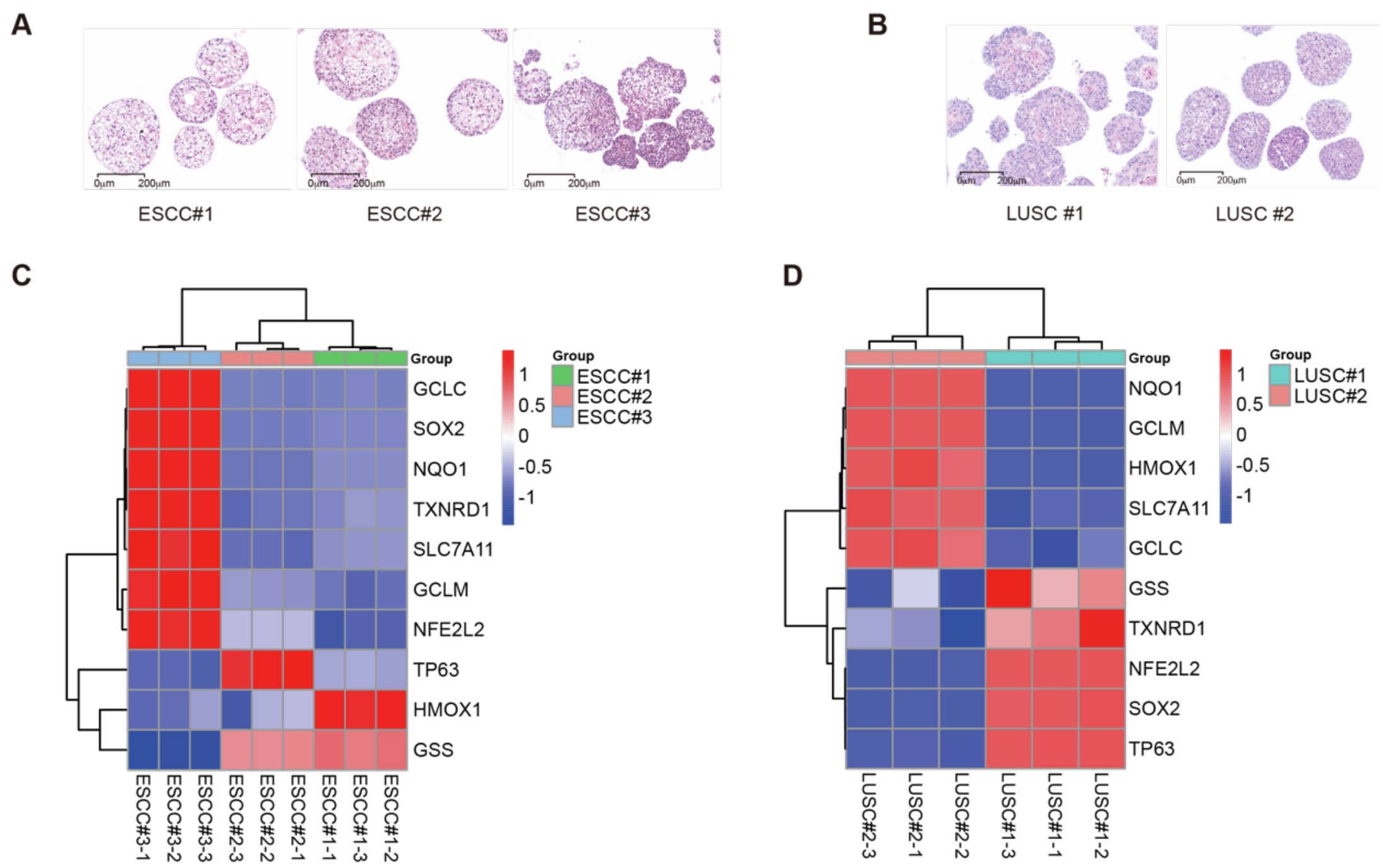

Figure S5. L013 suppresses viability of NRF2-hyperactivated organoids. Related to Figure 5. **(A, B)** H&E staining of ESCC and LUSC organoids. Scale bars represent 200 μm. **(C, D)** Heatmap of differentially expressed genes of NRF2 pathway in ESCC and LUSC organoids.

A

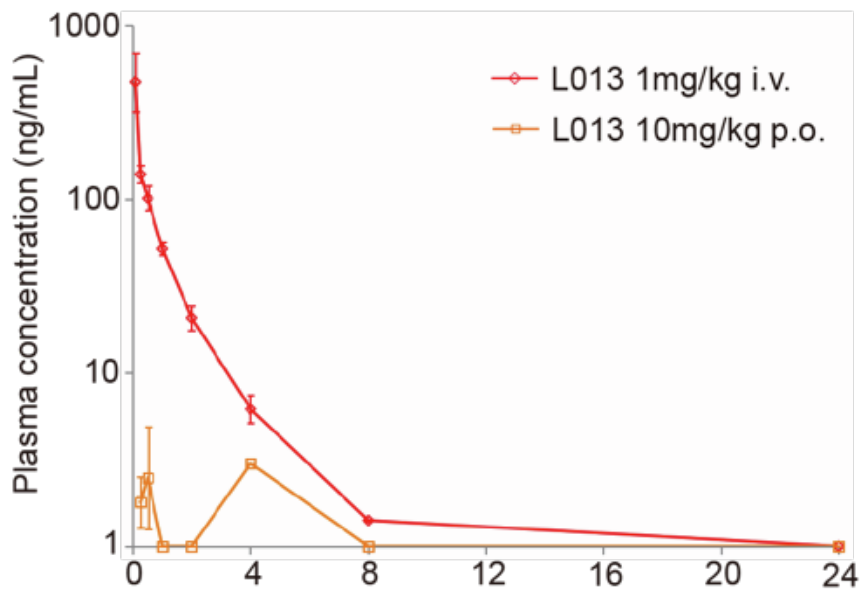

B

PK parameters	i.v. (1mg/kg)	p.o. (10mg/kg)
Cl_obs (mL/min/kg)	65.3	
$T_{1/2}$ (h)	1.6	NA
T_{max} (h)		2.1
C_0 (ng/mL)	944.7	
C_{max} (ng/mL)		8.7
AUC_{last} (h*ng/mL)	256.8	9.9
AUC_{Inf} (h*ng/mL)	260.1	NA
MRT_{Inf_obs} (h)	1.0	NA
$AUC_{last/D}$ (h*mg/mL)	256.8	1.0
Vss (L/kg)	4.1	
F (%)		0.4

C

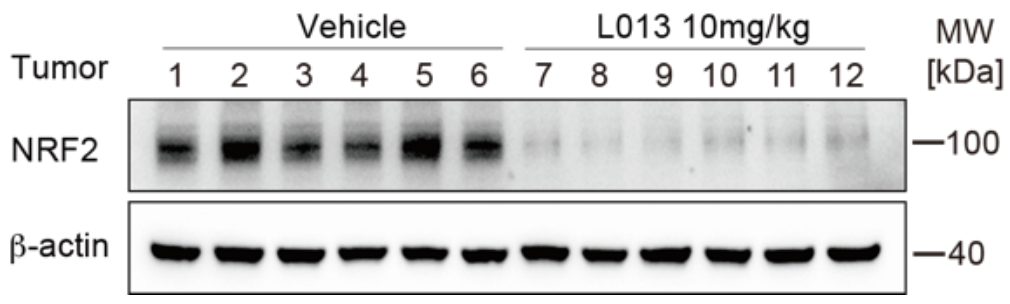

Figure S6. Pharmacokinetic study and PD effect of L013 in BALB/c nude mice. Related to Figure 6.

(A) Plasma concentration time curve of L013 with intravenous injection (i.v., 1 mg/kg) and oral administration (p.o., 10 mg/kg) in BALB/c nude mice (n = 3 per condition). **(B)** Pharmacokinetic parameters of L013 following intravenous (i.v., 1 mg/kg) and oral (p.o., 10 mg/kg) injection in BALB/c nude mice (n = 3 per condition). **(C)** Immunoblotting detection for NRF2 in BALB/c nude mice xenografts after 5 days treatment with L013 (p.o., 10 mg/kg, qd)

Supplementary Tables

Table S3. Primers for shRNAs, Related to STAR Methods

Primer name	Sequence (5' to 3')
shNRF2#1	GCTCCTACTGTGATGTGAAAT
shNRF2#2	TGGAGTAAGTCGAGAAGTA

Table S4. Primers for qPCR, Related to STAR Methods

Primer name	Sequence (5’ to 3’)
GAPDH	F: CCGGGAAACTGTGGCGTGATGG
	R: AGGTGGAGGAGTGGGTGTCGCTGTT
NFE2L2 (CDS)	F: AGGTTGCCCACATTCCCAAA
	R: ACGTAGCCGAAGAAACCTCA
NFE2L2 (5’UTR)	F: TTCTAGTTCGGACGCGGTG
	R: CCGCTCTGGGGAAAATAGCA
NRF1	F: GCTGATGAAGACTCGCCTTCT
	R: TACATGAGGCCGTTTCCGTTT
NRF3	F: TTCAGCCAGGCTATAAGTCAGG
	R: GCTCAGGATTGGTGGTATGAGA

Table S5. Primers for ChIP-qPCR, Related to STAR Methods

Primer name		Sequence (5’ to 3’)
NFE2L2 gene body-specific primers	P1	F: GACTGGGCTCTCGATGTGAC<br>R: CCCCTGTTGGTGGAAGACTC
	P2	F: AGGTAGATGGGTTGCACAGC<br>R: TCAGACACCAAGTGGCAGAC
	P3	F: CAGGTGGCTCAGATCTCCAC<br>R: ACGGTGTGAATGTGCTGGAT
	P4	F: CAGACCTACACCTTGGCAGG<br>R: GCAGGGCAGGTAAACTGGAT
NFE2L2 promoter-specific primers	P5	F: TCCGGGTGTGTTTGTTCCAA<br>R: AAATAAGGGCAGCGCTCTCG
	P6	F: TTCTCGGGCGGTAAAGTGAG<br>R: CTGGAGTTCGGACGCTTTGA
	P7	F: TTCAGGGTGACTGCGAACAC<br>R: ATCTCACTTTACCGCCCGAG
	P8	F: CTCCGACCTAAAGGGGCTTC<br>R: TTCGCAGTCACCCTGAACG

supplemental references

S1. Gyorffy B. Integrated analysis of public datasets for the discovery and validation of survival-associated genes in solid tumors. Innovation (Camb), 2024;5:100625.
